# Differential effects of pea protein isolate, pea protein concentrate, and whey protein isolate supplementation on gut microbiota and metabolic markers in healthy adults: a 3-arm parallel randomised intervention

**DOI:** 10.1007/s00394-026-04143-z

**Published:** 2026-11-11

**Authors:** Annalena Kamm, Samira Prado, Katharina Dannenberg, Dirk Repsilber, Tatiana M. Marques, Robert J. Brummer

**Affiliations:** 1 School of Medical Sciences, Faculty of Medicine and Health, Örebro University, Örebro, Sweden; 2 Food and Health Centre, Örebro University, Örebro, Sweden; 3 School of Science and Technology, Faculty of Business, Science and Engineering, Örebro University, Örebro, Sweden; 4 Clinical Genomics Örebro, Science for Life Laboratory, Örebro University, Örebro, Sweden

**Keywords:** *Anaerobutyricum hallii*, *Bifidobacterium longum*, Dietary intervention, Dietary proteins, *Lactobacillus crispatus*, *Limosilactobacillus frumenti*

## Abstract

**Purpose:**

As origin and quality of dietary protein are key modulators of gut microbiota and host metabolism, characterising health effects of different proteins is increasingly important given the need to shift towards more plant-based protein sources for environmental sustainability. Yet, direct comparisons across protein sources and food matrices in humans remain limited. This randomised, double-blind, 3-arm parallel, dietary intervention study examined the effects of four-week pea protein concentrate (PPC), pea protein isolate (PPI) or whey protein isolate (WPI) supplementation (0.5 g/kg body mass/day) on gut microbiota composition in healthy adults as the main outcome.

**Methods:**

Faecal samples and lifestyle questionnaires were collected weekly, with fasting blood and 24-h urine samples obtained at baseline, mid- and post-intervention. Proteins were compared using two contrasts – PPC vs. PPI and PPI vs. WPI – investigating differences between plant and animal-based proteins as well as between plant-based proteins with different matrixes.

**Results:**

Supplementation of PPC and PPI significantly reduced α-diversity and increased β-diversity, partly modulated by BMI. At species level, relative abundances of *Anaerobutyricum hallii* and *Bifidobacterium longum* increased significantly in subjects consuming PPC. A similar result was observed for *Lactobacillus crispatus* and *Limosilactobacillus frumenti* in subjects consuming PPI.

**Conclusion:**

Our findings demonstrate that different dietary proteins selectively modulate beneficial gut microbial species, highlighting the importance of investigating individual responses to dietary interventions. Further studies are needed to verify how compositional changes translate into changes in microbial functionality.

**Trial registration:**

This study is registered at www.clinicaltrials.gov as NCT05611138 (November 02, 2022).

**Supplementary Information:**

The online version contains supplementary material available at https://doi.org/10.1007/s00394-026-04143-z.

## Introduction

The origin of dietary protein has become an increasingly important focus of nutritional research in recent years. The growing interest is driven not only by the environmental advantages of plant-based proteins (PBPs), compared to animal-derived protein, but also by the expected health benefits associated with diets that include PBPs as the primary protein source [1, 2]. However, a major limitation of PBPs is their generally lower quality, characterised by reduced digestibility – partly due to antinutritional factors impairing digestive enzyme activity [3] – and a less favourable amino acid (AA) profile, both of which can affect their overall contribution to health [4].

Previous research has established that the gut microbiota plays a central role in mediating diet–health interactions [5]. Considerable attention has been devoted to dietary fibre, whose health benefits are largely mediated by microbial fermentation and the resulting production of short-chain fatty acids (SCFAs), supporting gastrointestinal and metabolic health [6, 7]. In contrast, the effects of different dietary protein sources on gut microbiota composition and associated health outcomes in humans remain comparatively understudied. Due to the lower digestibility of PBPs, a greater fraction reaches the colon, where undigested proteins and AAs become substrates for microbial fermentation [8]. In the presence of dietary fibre, gut microbes preferentially engage in saccharolytic fermentation. However, when fibre is depleted, proteins serve as the primary substrate, shifting fermentation towards proteolytic pathways which increase the production of potentially harmful metabolites such as ammonia, hydrogen sulphide, and phenolic compounds [9]. Nevertheless, the specific metabolites produced depend not only on the available substrates but also on the microbial species residing in the gut and their enzymatic activity [10, 11].

Previous studies investigating the effects of dietary proteins on gut microbiota composition have demonstrated that different protein sources can distinctly shape microbial community and function [12–15]. Although most of this research has been conducted in rodent models, findings indicate that both the quantity and source of dietary protein affect the observed microbial responses [12]. These effects likely arise from variations in protein digestibility, determining the amount of fermentable protein in the colon, as well as from differences in AA composition further modulating substrate availability [12].

To improve the understanding of the differential health effects of protein sources, this dietary intervention study directly compares supplementation with pea protein isolate (PPI) to whey protein isolate (WPI). To further assess the role of the food matrix within a single protein source, effects of pea protein concentrate (PPC) and PPI supplementation are compared. Pea protein was selected as legumes generally have a higher protein content than grains and are well suited for environmentally sustainable cultivation in the Nordic region [16]. Furthermore, the same pea protein has been used in previous dietary intervention and physicochemical characterisation studies conducted, providing an established basis for its use in the present study [17]. The main outcome of this study is the change in gut microbiota composition. Additionally, potential broader health impacts are assessed as secondary outcomes by measuring a range of biological markers, including glucose metabolism and inflammatory markers as well as blood lipids.

## Materials and methods

### Study design and recruitment

This randomised, double-blind, 3-arm parallel, dietary intervention study was conducted between September 2022 and April 2023 at Örebro University, Sweden. The study was approved by the Swedish Ethical Review Authority (Dnr 2021-03256, 2022-04793-02), conducted in accordance with the Declaration of Helsinki, and registered on www.clinicaltrials.gov (NCT05611138). Study subjects were recruited by advertisements on social media and the university’s webpage as well as posters placed in public areas in the city of Örebro. Before the start of the study, eligibility of participants was assessed during a screening visit. Interested subjects were eligible to participate if they fulfilled all required inclusion criteria and did not meet any of the study’s exclusion criteria before enrolment (Online Resource 1). Before any study-related activity, a written informed consent was obtained.

**Fig. 1:**
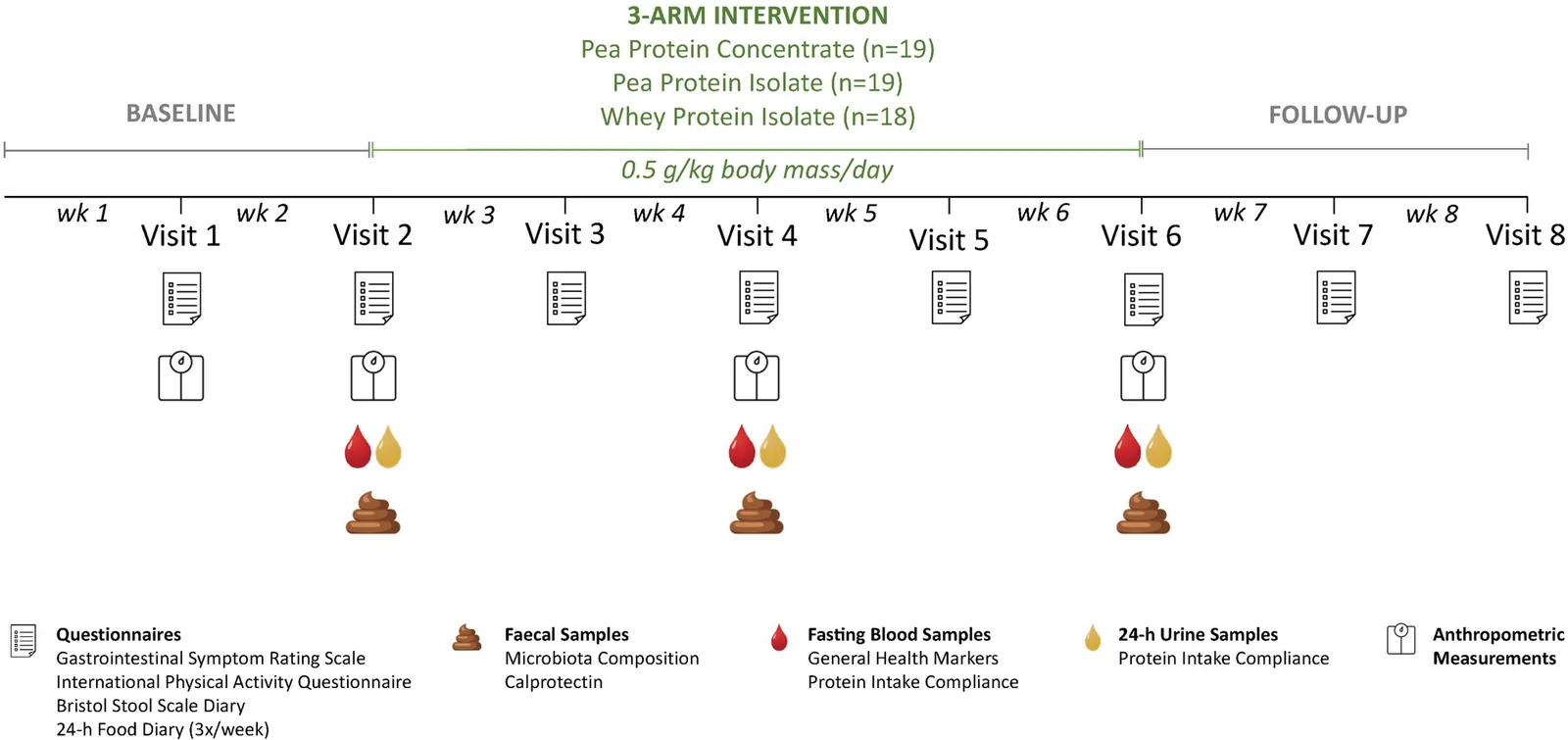
Overview of the randomised, double-blind, 3-arm parallel, dietary intervention study. The study comprised a two-week baseline period, a four-week intervention period, and a two-week follow-up period, with study visits scheduled at one-week intervals. During the intervention period, participants consumed one of the tested protein supplements at an amount standardised to body mass, divided into three equal portions and distributed throughout the day; wk: week

Subsequently, participants were randomised to one of the three study arms and scheduled for eight study visits (V1-V8), taking place one week apart. The study comprised a two-week baseline period, a four-week intervention period, and a two-week follow-up period. On all visits, participants were asked to hand in completed questionnaires assessing dietary habits, physical activity, gastrointestinal symptoms as well as bowel habits. During the first visit, participants were weighed to calculate the amount of protein they were going to supplement for four weeks starting at V2 (intervention baseline). The visits during the intervention period were scheduled in the morning after a fasting period of at least 10 h, during which blood samples as well as 24-h urine samples and faecal samples were collected from the participants. In addition, body mass and height were assessed (Fig. 1).

### Study outcomes

The primary outcome is defined as the change in gut microbiota composition in faecal samples collected before and after the intervention. Secondary outcomes include changes in surrogate health markers in faecal, blood, and urine samples, lifestyle changes, including diet and physical activity, as well as the development of gastrointestinal symptoms during the study.

### Study product

Participants supplemented either PPC (P6511C, Vestkorn), PPI (Pisane C9, Cosucra), or WPI (Protura Sweet, Toft Care) (Table 1). All products were commercially available and the PPC and PPI used in the present study have been well characterised in the literature before [17]. Whey protein isolate is a frequently used animal-based control applied in dietary intervention studies.

**Table 1:** Nutritional intake from the study products according to product specifications calculated for an individual with a body mass of 70 kg

	PPC	PPI	WPI
Energy (kcal)	210.0	149.9	146.3
Carbohydrates (g)	5.9	0.3	0.2
- Sugar (g)	0.8	0.0	0.2
- Dietary Fibre (g)	5.4	1.0	0.0
Fat (g)	3.2	0.7	0.7
- Saturated (g)	0.5	0.1	0.4
Protein (g)	35.0	35.0	35.0
Sodium (g)	0.6	0.6	0.5

*PPC* Pea Protein Concentrate, *PPI* Pea Protein Isolate, *WPI* Whey Protein Isolate

The total amount of protein powder per day was individually calculated based on baseline body mass (0.5 g/kg body mass/day) and divided into three equal portions. The dose was chosen to achieve – together with the participants’ habitual diet – a higher, but still physiologically relevant protein intake of > 1.0 g/kg body mass/day. This decision was also based on the results of a previously conducted study, in which first changes of relevant biomarkers and metabolites were observed after supplementing with 0.5 g of PPI/kg body mass/day [18].

Participants were instructed to maintain their habitual diet and consume each portion together with breakfast, lunch, and dinner to ensure an even daily intake and avoid high protein loads. The supplement powders could be consumed mixed with a drink or food product of the participants’ choice. No specific instructions or recipes were provided to avoid introducing new food products into their habitual diet. Participants were asked to return all received protein bags, regardless of whether or not they were consumed, to assess compliance later on.

### Biological samples

Fasting blood samples were collected in serum and plasma tubes (Becton Dickinson, United Kingdom), using a venipuncture set (Greiner Bio-One GmbH, Austria), and subsequently centrifuged according to the manufacturer’s instructions. The supernatant was aliquoted and stored at -80 °C until analysis of insulin in serum as well as triglycerides, total cholesterol, high-density lipoprotein (HDL) cholesterol, low-density lipoprotein (LDL) cholesterol, creatinine, urea, and C-reactive protein (CRP) in plasma. Glucose levels were analysed from whole blood collected in a separate tube (Becton Dickinson, United Kingdom) directly after the study visit. All analyses were performed in the Clinical Chemistry Department at Örebro University Hospital according to automated and standardised procedures.

Urine samples were collected over 24 h in a designated container (Sarstedt, Germany). Participants were instructed to start the collection in the morning on the day before the study visit after discarding the morning urine and to end the collection in the morning on the day of the study visit including the morning urine. The container was stored at 6–8 °C between sampling occasions and missed occasions resulted in exclusion of the data. Well-homogenised aliquots were collected from the container with vacuum tubes (Sarstedt, Germany), stored at -20 °C during the study visits, and thereafter transferred to -80 °C until analysis for creatinine, urea, and uric acid. Analyses were performed in the Clinical Chemistry Department at Örebro University Hospital applying automated and standardised procedures.

Faecal samples were collected on the day of the study visit or one day before using an EasySampler Stool Collector Kit (GP Medical Devices, Denmark) and faecal sample collection tubes (Sarstedt, Germany). If collected at home, tubes were stored at -20 °C and samples were transported to the study centre in a designated frozen transport container (Sarstedt, Germany). At the study centre, the tubes were stored at -20 °C during the study visit and further transferred to -80 °C until analysis. After thawing, CALEX cap tubes (BÜHLMANN, Switzerland) were used for extracting aliquots for faecal calprotectin analysis at the Clinical Chemistry Department at Örebro University Hospital using automated and standardised procedures.

Microbiota analysis of faecal samples was performed using samples collected at V2, V4, and V6 at Clinical Genomics, Örebro using established protocols described elsewhere [18]. Briefly, aliquots were collected from each sample, DNA was extracted, purified, and quantified according to the manufacturer’s instructions using the QIAsymphony PowerFaecal Pro DNA Kit (Qiagen, Germany), the QIAsymphony SP liquid handler (Qiagen, Germany) and the Qubit 2.0 fluorometer (Thermo Fisher, USA). Next-generation sequencing was performed using the NextSeq 2000 sequencer (Illumina, USA) and samples were randomised across sequencing runs to minimise batch effects. Subsequently, data were processed with default settings of the taxprofiler pipeline (version 1.1.2; nextflow version 24.10.0) [19] using the MetaPhlAn (4.0.6) taxonomic profiler.

### Lifestyle and symptom questionnaires

To assess changes in participants’ habitual dietary intake during the study regarding total energy and macronutrient intake, each participant completed three 24-h food diaries per week. These diaries recorded food consumption on two weekdays and one weekend day of the participant’s choice, collected on non-consecutive days. During their initial screening visit, all participants received training on how to properly fill in their diaries. As additional support, they received a guide with pictures of different portion sizes for all main food groups and an information brochure with additional instructions. The food diary and the guide were provided by the Swedish Food Agency [20].

Physical activity and lifestyle maintenance during the study were documented using the International Physical Activity Questionnaire (IPAQ), which includes questions regarding different physical activity intensities during the past seven days, such as walks, medium, and high-intensity activities [21]. The questionnaire was completed on the evening before each visit. With the help of the IPAQ scoring guidelines, answers were converted into total metabolic equivalent (MET)-min/week and a physical activity level (PAL) ranging from 1 (low) to 3 (high) [22].

To monitor gastrointestinal symptoms during the study, participants also filled in a Gastrointestinal Symptom Rating Scale (GSRS) containing 15 questions regarding various gastrointestinal symptoms in a seven-point graded Likert-type scale on the evening before each study visit [23]. The questionnaire covers symptoms occurring during the past seven days and scores range from 1, representing no discomfort, to 7, representing very severe discomfort. In addition, the frequency and stool consistency of bowel movements were documented using the Bristol Stool Scale (BSS) ranging from 1 (hard) to 7 (liquid) in a BSS diary [24].

### Statistical analyses

The power calculation was conducted using G*Power (Version 3.1.9) based on the primary outcome to investigate within-participant microbiota composition changes in faecal samples obtained before and after the dietary intervention. As a planning benchmark for the magnitude of change we accounted for the fact that diet can contribute to 44% of total variation of gut microbiota composition [25]. We specified a conservative standardised within-participant effect size (standardised mean paired difference; Cohen’s *d*_*z*_ of 0.6, informed by the magnitude of change reported in Johnson et al. (1.24 SD) and assuming approximately half the effect). The sample size was estimated using a Wilcoxon signed-rank test (matched pairs), two-sided α = 0.05 with the aim of reaching a power of 90%. Under these assumptions, and when including a dropout rate of 20%, 29 subjects were required in each arm to reach the anticipated power.

Subsequent analyses of collected data sets were conducted in R (Version 4.4.1) using RStudio (Version 2024.04.2 + 764), applying a significance level of α = 0.05 and two contrasts – WPI vs. PPI, comparing the effects of protein isolates from different sources, as well as PPI vs. PPC, investigating the effects of different matrixes originating from the same source.

Prior to the main analysis, baseline differences in age, BMI, and sex were assessed using the Mann-Whitney U test and the Fisher’s exact test. Additionally, outliers in the biomarker dataset, defined as values outside ± 3 SD from the mean, were removed. In the BSS diary data analysis, one participant was excluded due to missing data. Information on the extent of the final data set is included in the corresponding data tables. Additionally, data were log-transformed and assessed for normal distribution applying the Shapiro-Wilk test as well as by inspecting QQ-plots.

For microbiota data, counts were scaled to percentages (sum = 100) per sample, and a sparsity reduction was conducted by removing all taxa whose sum across samples was within the bottom 30% of sums, as well as all taxa that appeared in fewer than two samples. Subsequently, data were log-transformed (log1p(x)) and standardised to a mean of zero and a standard deviation of one for each taxon across samples. Only participants with complete datasets were included in analyses at species level, resulting in the exclusion of six participants. α-diversity was assessed using the Shannon Diversity Index and calculated at the sample level from OTU count data, while β-diversity was quantified using the Bray-Curtis Index by calculating pairwise distances for each participant between samples from V2 to V4 as well as V2 to V6 (vegan package). 

Non-normally distributed data were analysed fitting a nonparametric longitudinal model (nparLD) with time (V2, V4, and V6) and treatment (intervention group: PPC, PPI, and WPI) as factors, including time:treatment interactions. Post-hoc multiple comparisons investigated specific differences over time within each intervention group by comparing V4 and V6 to V2. Specific differences between treatments were investigated at each visit and were performed by employing Mann-Whitney U tests (rstatix package) with Benjamini–Hochberg correction for multiple testing. To target downstream analyses of the large microbiota data set, post-hoc multiple comparisons focused on species with significant interaction terms time:treatment and time:treatment:BMI. Benjamini-Hochberg correction of p-values was conducted after filtering for the above-mentioned interaction terms, before conducting post-hoc multiple comparisons. Data fulfilling the assumptions for parametric analyses were analysed using linear mixed-effects models (LMMs) from the nlme package and base R. The LMMs included the same fixed effects as mentioned above, with random intercepts for each participant. Post-hoc multiple comparisons were conducted following the same principle as mentioned above using estimated marginal means (EMMs) derived from the fitted model, comparing WPI vs. PPI as well as PPI vs. PPC using the emmeans package. Resulting p-values were adjusted for multiple testing using the Sidak method (food diaries) and Benjamini-Hochberg correction (microbiota diversity measures).

Biomarker and microbiota analyses included the additional term BMI as an exploratory analysis of potential subgroup-specific effects [26–28]. Participants were stratified into two BMI groups (≤ 23.2 and > 23.2 kg/m^2^) based on the global median BMI of the study population at baseline, which achieved the most equal distribution of participants (PPI: 12:7, PPC: 7:12, WPI: 9:9). Post-hoc multiple comparisons only considered BMI-groups if the main model yielded significant results for the factor BMI or when BMI was included in a significant interaction term.

Compliance with the intervention was assessed based on the number of returned, empty protein bags using the Mann-Whitney U test in GraphPad Prism (Version 10.6.1 (892)).

## Results

In total, 60 participants were enrolled in the intervention phase of the study, with four dropping out due to personal reasons, including the taste of the supplement, or due to fulfilling one of the exclusion criteria after the start of the study. Due to the per-protocol analysis approach, only the 56 subjects completing the study were included in the subsequent analyses (Online Resource 2). At baseline, the three intervention groups did not differ significantly in age, BMI, or sex distribution (Table 2).

**Table 2:** Participant’s baseline characteristics at time of inclusion (*N* = 56 in all three arms)

	PPC		PPI		WPI		*p*-value PPC vs. PPI	*p*-value PPI vs. WPI
	Median (IQR)	*n*	Median (IQR)	*n*	Median (IQR)	*n*		
Age *(years)*								
Total	29.0 (21.0–35.5)	19	31.0 (22.0–42.0)	19	26.5 (22.0–34.8)	18	0.3718	0.4025
Female	22.0 (19.0–33.0)	13	29.0 (21.0–41.0)	17	25.5 (21.8–35.0)	16	0.2233	0.5759
Male	34.0 (31.0–37.8)	6	38.5 (36.2–40.8)	2	31.0 (29.0–33.0)	2	0.4918	0.6667
BMI *(kg/m*^*2*^*)*								
Total	23.3 (22.5–24.6)	19	22.8 (21.1–24.5)	19	23.2 (20.8–24.2)	18	0.1887	0.8079
Female	23.2 (22.4–23.7)	13	22.3 (21.0–24.4)	17	23.1 (20.6–24.5)	16	0.3049	0.6786
Male	24.6 (24.2–25.6)	6	25.4 (24.5–26.2)	2	23.5 (23.3–23.7)	2	0.8571	0.6667

Significant differences were evaluated using the Mann-Whitney U test and the Fisher’s exact test; *IQR* Interquartile Range, *PPC* Pea Protein Concentrate, *PPI* Pea Protein Isolate, *WPI* Whey Protein Isolate, α = 0.05

### Gut microbiota composition

Microbiota composition was assessed by investigating changes in α-diversity (Shannon Diversity Index) and β-diversity (Bray-Curtis Index), as well as by characterising taxonomic composition at the species level, including profiling the most abundant phyla and evaluating changes in species-level composition over the course of the intervention.

Descriptive statistics of gut microbiota diversity measures including the results of the LMMs are summarised in Online Resource 3. Briefly, α-diversity differed significantly over time as well as between BMI groups in both comparisons (PPC vs. PPI and PPI vs. WPI). Differences between the interventions (treatment) were only observed when comparing PPC vs. PPI. β-diversity (Bray-Curtis Index) differed significantly over time in both comparisons PPC vs. PPI and PPI vs. WPI. To characterise the nature of the observed differences, post-hoc multiple comparisons were performed. As BMI was a significant factor in the main model assessing changes in α-diversity, BMI grouping was also incorporated into the corresponding post-hoc analyses. In contrast, post-hoc multiple comparisons for β-diversity were performed without stratification by BMI.

When comparing the PPC and PPI-arm, α-diversity was significantly lower in participants consuming PPI in the higher BMI-group (> 23.2 kg/m^2^) at V2 (*p* = 0.0150) and V6 (*p* = 0.0370) compared to those in the lower BMI-group (≤ 23.2 kg/m^2^). Similarly, α-diversity within the PPC-arm was significantly lower at all visits – V2, V4, and V6 – in the higher compared to the lower BMI group (*p* = 0.0022, 0.0067 and 0.0021, respectively). Furthermore, α-diversity differed significantly over time in subjects belonging to the lower BMI-group supplementing PPI and was significantly lower at V6 compared to V2 (*p* = 0.0089). α-diversity was also significantly lower at V6 compared to V2 in subjects supplementing PPC, however, only in those belonging to the lower-BMI group (*p* = 0.0156).

**Fig. 2:**
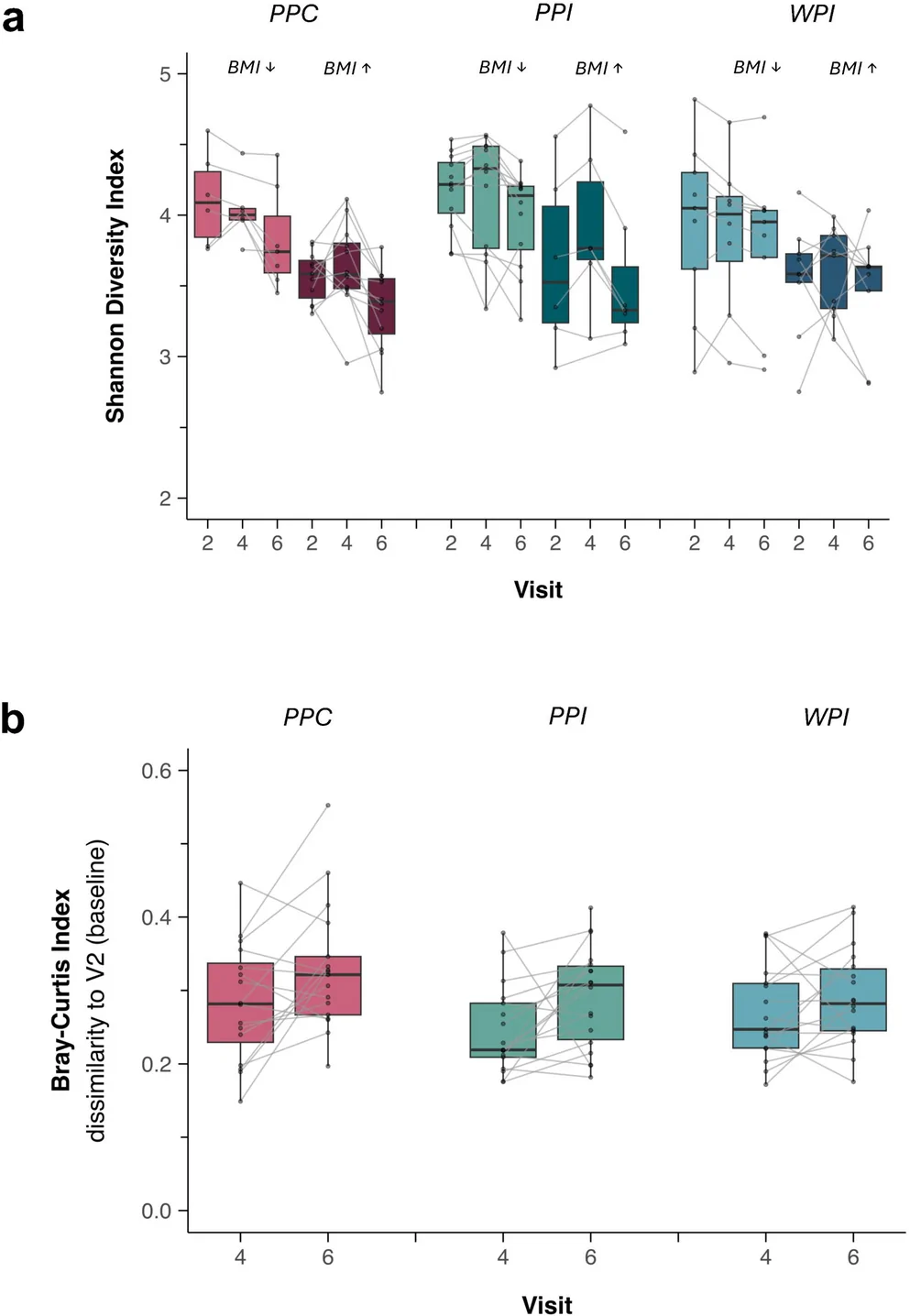
Change of gut microbiota diversity measures over the course of the intervention. **a** Shannon Diversity Index (α-diversity) over the course of the intervention, grouped by BMI and calculated at the sample level from OTU count data, (**b**) Bray-Curtis Index (β-diversity) over the course of the intervention, quantified by calculating pairwise distances for each participant between samples from V2 to V4 (V4) as well as V2 to V6 (V6); BMI ↓: ≤ 23.2 kg/m^2^, BMI ↑: > 23.2 kg/m^2^; PPC: Pea Protein Concentrate, PPI: Pea Protein Isolate, V: Visit, WPI: Whey Protein Isolate

When comparing the PPI and WPI-arm, post-hoc analyses revealed the same results during PPI supplementation as described above. No significant differences in α-diversity were observed within the WPI-arm as well as between the arms (Fig. 2a).

Multiple comparisons investigating specific differences in β-diversity revealed no significant differences between the arms. However, dissimilarity was significantly greater between the samples collected at V6 and V2 compared to the samples collected at V4 and V2 within the PPI (PPI vs. PPC: *p* = 0.0346, PPI vs. WPI: *p* = 0.0213) and the PPC-arm (*p* = 0.0322). No significant differences in β-diversity over the course of the intervention were observed within the WPI-arm (Fig. 2b).

**Fig. 3:**
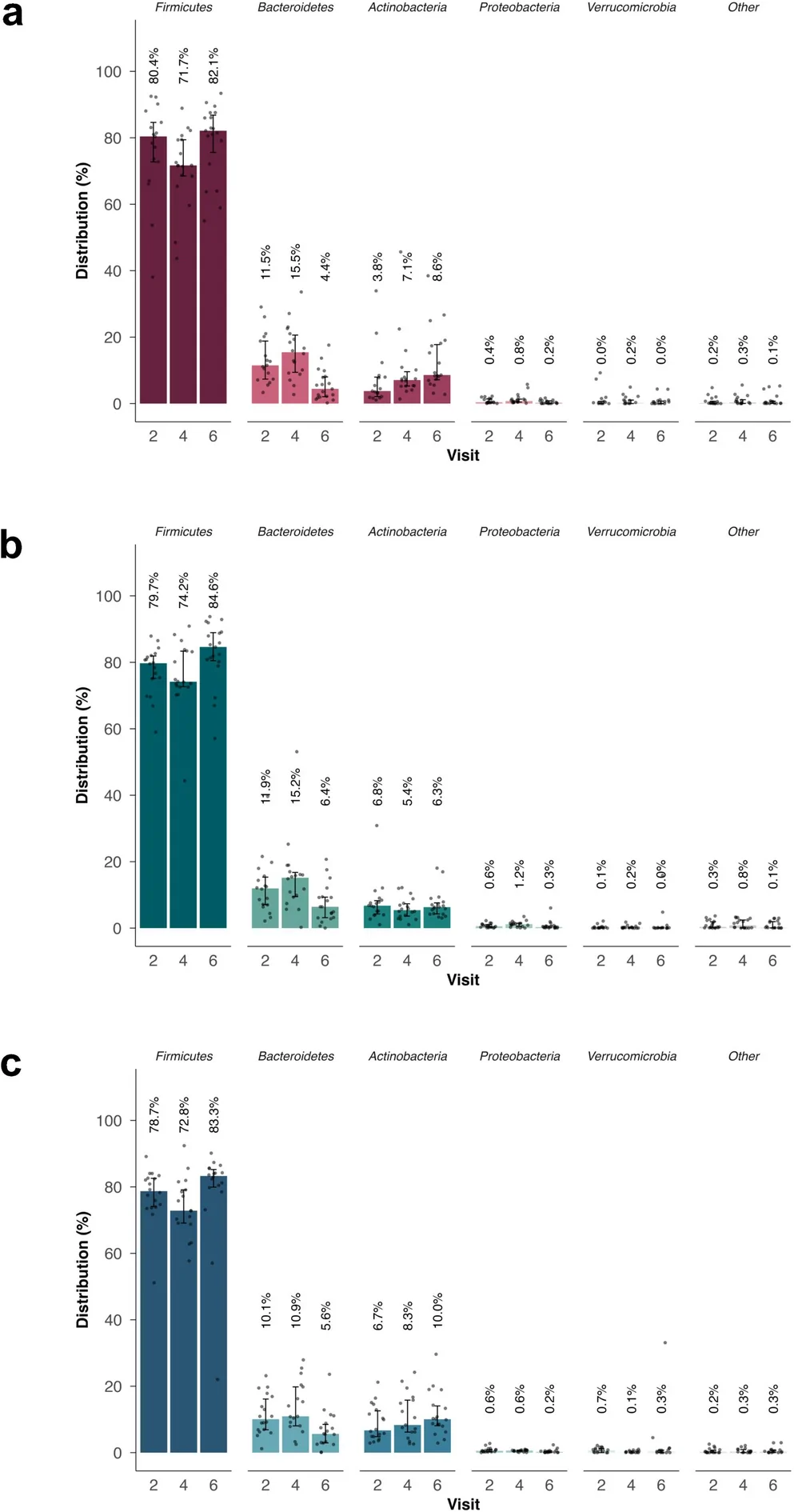
Gut microbiota composition of participants in each of the intervention arms. Distribution (%) including IQR of phyla calculated for each participant and summarised over the whole population consuming PPC (**a**), PPI (**b**) and WPI (**c**); IQR: Interquartile Range, PPC: Pea Protein Concentrate, PPI: Pea Protein Isolate, WPI: Whey Protein Isolate

The most dominant phyla were Firmicutes, followed by Bacteroidetes and Actinobacteria, collectively comprising over 94% of detected species across all intervention groups and visits (Fig. 3). An additional overview of the species that are amongst the top 10 most abundant per participant and visit is provided in Online Resource 4.

When investigating the effects of PPC vs. PPI supplementation at the species level, 261 out of 990 species demonstrated significant differences for at least one of the main factors time, treatment, BMI, as well as their interactions in the main model. To reduce the amount of multiple comparisons and to increase the meaningfulness of the results, species were prefiltered based on significant descriptive p-values for the interaction terms time:treatment (*n* = 22) and time:treatment:BMI (*n* = 21) before conducting post-hoc analyses (Online Resource 5). The same approach was applied when comparing the two protein isolates (PPI vs. WPI). 277 species differed regarding at least one of the factors time, treatment, BMI, and their interactions in the main model. Further filtering for significant interaction terms resulted in 36 species with significant time:treatment interactions and 12 species with significant time:treatment:BMI interactions. Subsequent BH-adjustment before conducting multiple comparisons did not affect the number of significant species (Online Resource 6). Species with significant results (BH-adjusted) in the multiple comparisons are presented in more detail in the following.

**Fig. 4:**
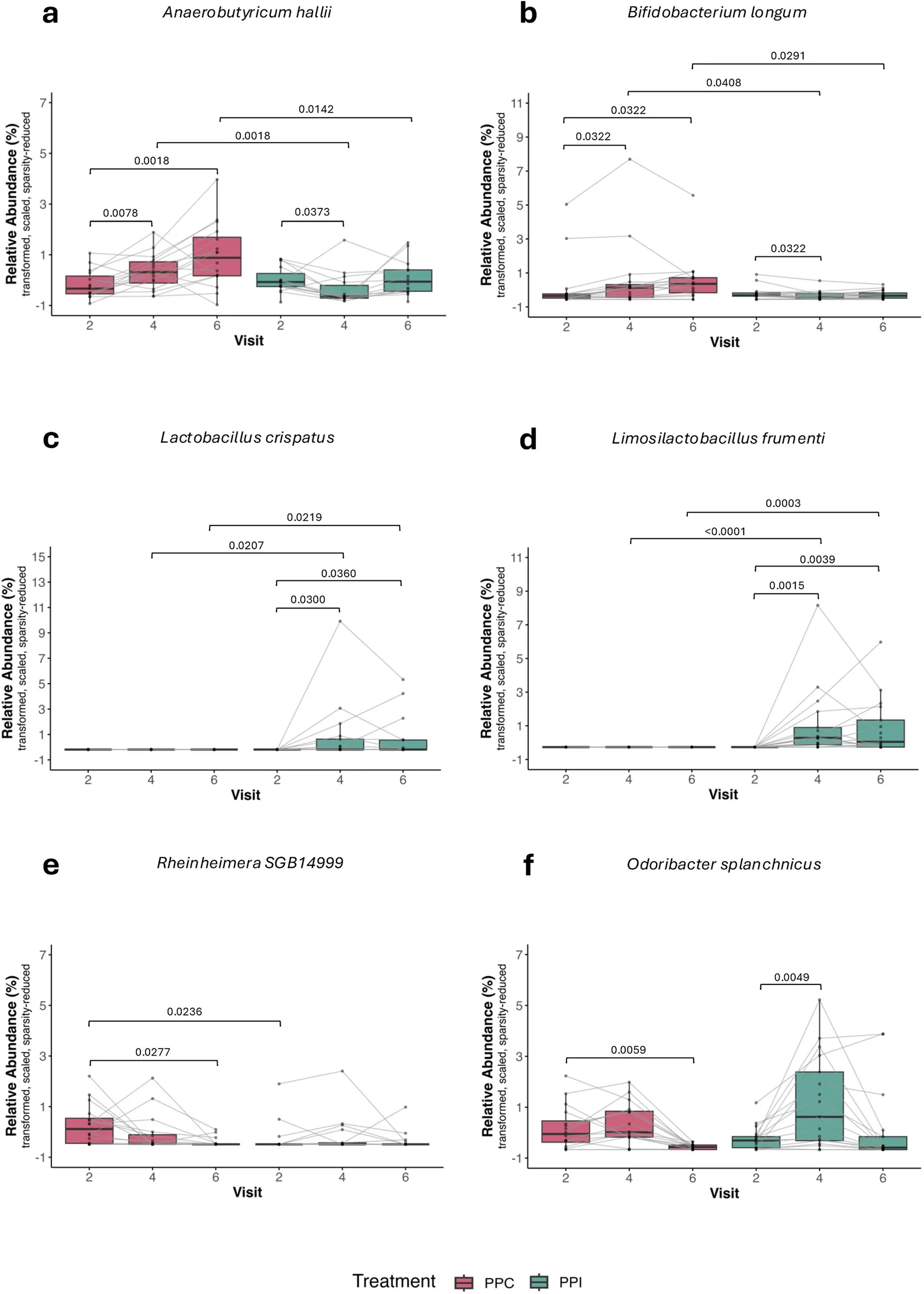
Relative abundance (%) of species with significant time:treatment interactions determined by LMMs comparing PPC vs. PPI. Graphs visualise median (IQR) of log-transformed, scaled, and sparsity reduced relative abundances including p-values obtained from the post-hoc multiple comparisons (Benjamini–Hochberg adjusted). Sparsity reduction was performed by removing all taxa whose sum across samples was within the bottom 30% of sums, as well as all taxa that appeared in fewer than two samples. Data were log-transformed using log1p(x) and standardised to a mean of zero and a standard deviation of one for each taxon across samples; IQR: Interquartile Range, LMMs: Linear mixed-effect models, PPC: Pea Protein Concentrate, PPI: Pea Protein Isolate, α = 0.05

We first conducted post-hoc analyses for species with significant time:treatment interactions comparing PPC vs. PPI. Differences between treatments were observed for *Anaerobutyricum hallii* and *Bifidobacterium longum* with significantly higher relative abundances at V4 and V6 in the PPC-arm compared to the PPI-arm (Fig. 4a and b). In contrast, relative abundances of *Lactobacillus crispatus* and *Limosilactobacillus frumenti* were significantly higher at V4 and V6 in the PPI-arm compared to the PPC-arm (Fig. 4c and d). Furthermore, we observed baseline differences between the intervention arms for *Rheinheimera SGB14999* (Fig. 4e) and *Flavonifractor plautii* (graph not shown) with significantly higher relative abundances at V2 in the PPC-arm. Relative abundances of *Clostridium sp NSJ 42* were significantly lower at V4 in the PPC-arm (graph not shown). Changes over time within an intervention group were observed for *Anaerobutyricum hallii* and *Bifidobacterium longum* for which relative abundances significantly increased at V4 and V6 compared to V2 in the PPC-arm (Fig. 4a, b). Relative abundances of *Lactobacillus crispatus* and *Limosilactobacillus frumenti* increased as well with the same pattern, however, these changes were observed in people supplementing PPI (Fig. 4c and d). In contrast, relative abundances of *Rheinheimera SGB14999* decreased significantly over the course of the intervention in the PPC-arm (Fig. 4e).

Relative abundances of *Odoribacter splanchnicus* were significantly lower when comparing V6 to V2 within the PPC-arm and significantly higher at V4 compared with V2 within the PPI-arm (Fig. 4f).

Post-hoc analysis for significant 3-way interactions yielded significant results for *Lacrimispora amygdalina* and *GGB74463 SGB54347* in the lower BMI-group. Within this group, relative abundances of *Lacrimispora amygdalina* were significantly higher at V4 compared to V2 during PPI supplementation (*p* = 0.0068). For *GGB74463 SGB54347* we observed higher abundances at V4 in the PPC-arm compared to the PPI-arm (*p* = 0.0499) (graphs not shown).

**Fig. 5:**
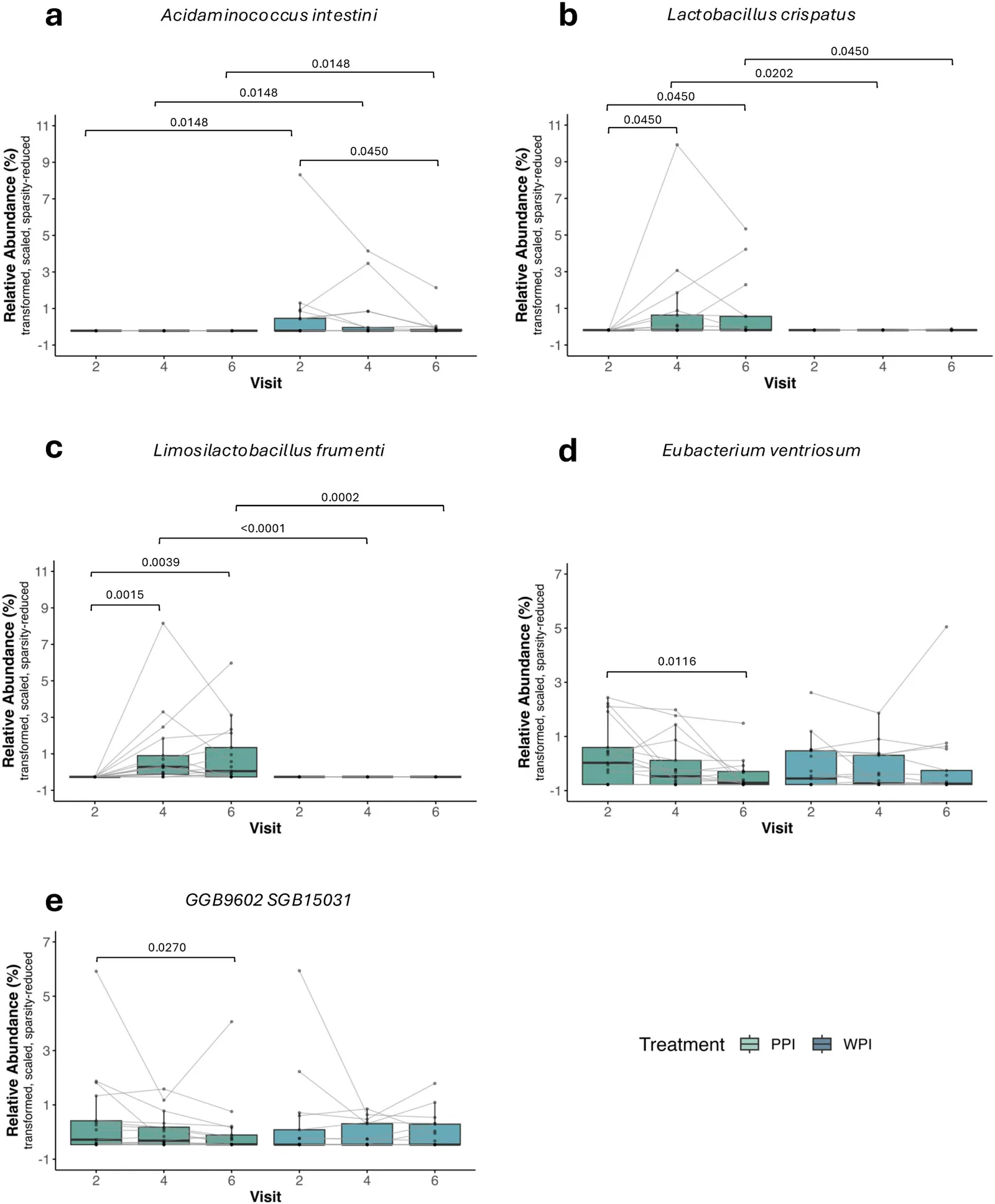
Relative abundance of species with significant time:treatment interactions determined by LMMs comparing PPI vs. WPI. Graphs visualise median (IQR) of log-transformed, scaled, and sparsity reduced relative abundances including p-values obtained from the post-hoc multiple comparisons (Benjamini–Hochberg adjusted). Sparsity reduction was performed by removing all taxa whose sum across samples was within the bottom 30% of sums, as well as all taxa that appeared in fewer than two samples. Data were log-transformed using log1p(x) and standardised to a mean of zero and a standard deviation of one for each taxon across samples; IQR: Interquartile Range, PPI: Pea Protein Isolate, WPI: Whey Protein Isolate, α = 0.05

Subsequently, we conducted post-hoc analyses for species with significant time:treatment interactions comparing PPI vs. WPI. Relative abundances between the intervention arms differed significantly for *Acidaminococcus intestini* with significantly lower abundances at all visits (V2–V6) in the PPI-arm compared to the WPI-arm (Fig. 5a). Additionally, relative abundances of *Lacrimispora saccarolytica* were significantly lower at V4 in the PPI-arm (graph not shown; *p* = 0.0443) and at V6 we observed significantly lower abundances of *Ruthenibacterium lactatiformans* (graph not shown; *p* = 0.0422). In contrast, relative abundances of *Lactobacillus crispatus* and *Limosilactobacillus frumenti* were significantly higher at V4 and V6 in the PPI-arm compared to the WPI-arm (Fig. 5b and c, respectively). Furthermore, *Eubacteriales unclassified SGB15145*, *GGB4768 SGB6601*, *GGB9522 SGB14921*, and *GGB9634 SGB15093* exhibited significantly higher relative abundances at V4 (graphs not shown; *p* = 0.0007, *p* = 0.0212, *p* = 0.0278 and *p* = 0.0114, respectively). At V6, we observed higher relative abundances of *Eubacteriales unclassified Clostridiales bacterium KLE1615* in the PPI-arm compared to the WPI-arm (graph not shown; *p* = 0.0368).

Changes over time within an intervention group were observed for *Acidaminococcus intestini* within the WPI-arm, showing significantly reduced relative abundances at V6 compared to V2 (Fig. 5a). Within the PPI-arm, relative abundances of *Lactobacillus crispatus* and *Limosilactobacillus frumenti* were significantly higher at V4 and V6 compared to V2 (Fig. 5b and c, respectively). In contrast, relative abundances of *Eubacterium ventriosum* and *GGB602 SGB15031* were significantly lower at V6 compared to V2 (Fig. 5d and e, respectively).

No post-hoc test for species with significant 3-way interactions reached statistical significance.

### Dietary habits, body mass, and physical activity

Most participants followed an omnivorous diet, while only five were vegetarian (PPI: *n* = 3, WPI: *n* = 2). Original, non-transformed data of participants’ dietary characteristics as reported in the 24-h food diaries as well as anthropometric measurements are summarised using median (IQR) in Online Resource 7, including statistical results of the main analysis for each factor time, treatment, and their interactions. Briefly, time effects for almost all variables in both comparisons (PPC vs. PPI and PPI vs. WPI) were significant except for dietary fibre (g/day) and fat (E%) intake when comparing PPI vs. WPI. In this comparison, however, treatment effects were significant for protein intake (g/kg body mass/day). No significant interactions were observed. To specify the direction of the observed differences, post-hoc analyses were performed.

Multiple comparisons investigating differences between the PPC and PPI-arm revealed significantly increased protein intake (g/kg body mass/day and E%) within both arms when comparing V4 and V6 to V2 (all *p* < 0.0001). Within the PPI-arm the intake of carbohydrates (E%) decreased significantly during the intervention resulting in significantly lower consumption at both V4 and V6 compared to V2 (*p* = 0.0170 and 0.0118, respectively). During PPC supplementation, the intake of carbohydrates was significantly lower at V4 compared to V2 (*p* = 0.0072). In addition, fat consumption (E%) was significantly lower in these subjects at V6 compared to V2 (*p* = 0.0061) while the intake of dietary fibre (g/d) increased when comparing both V4 and V6 to V2 (*p* = 0.0170 and 0.0084, respectively). Post-hoc tests did not reveal any significant differences in energy intake.

When comparing the intervention arms PPI and WPI, we observed an increased protein intake (g/kg body mass/day and E%) within both arms when comparing V4 and V6 to V2 (all *p* < 0.0001). The intake of carbohydrates (E%) decreased significantly during the study and was lower within both arms at V4 and V6 compared to V2 (PPI: *p* = 0.0206 and 0.0145, respectively; WPI: *p* = 0.0236 and 0.0311, respectively). Post-hoc tests for energy intake (kcal/d) did not reach statistical significance. Protein intake measured in g/kg body mass/day differed significantly at V2 between the two arms and was higher in subjects in the PPI-arm (*p* = 0.0207).

When analysing participants’ compliance based on the amount of returned, empty protein bags, results revealed high compliance with median (IQR) percentages of 98.8% (96.4–100.0%) in the PPC-arm, 95.2% (88.1–100.0%) in the PPI-arm, and 98.2% (90.2–100.0%) in the WPI-arm. The observed compliances did not differ significantly between the arms.

Post-hoc analysis of anthropometric data revealed that body mass as well BMI increased significantly in the PPI-arm when comparing V6 to V2 in both contrasts PPC vs. PPI and PPI vs. WPI (Body mass: *p* = 0.0011 and 0.0025, BMI: *p* = 0.0124 and 0.0115).

Physical activity measured in total MET minutes/week and PAL remained stable throughout the intervention (Online Resource 8).

### Gastrointestinal symptoms and bowel habits

Gastrointestinal symptom rating scores were generally low and ranged between 1 and 3.5 (IQR). The stool type and frequency ranged from 2.8 to 4.9 (IQR) and 0.7 to 1.7 (IQR). Results of the main statistical model are summarised in Online Resources 9 (GSRS) and 10 (stool type and frequency). Subsequently conducted multiple comparisons did not reveal any significant differences between the arms for any of the GSRS (sub-)scores. However, within the PPC-arm, indigestion subscores were significantly higher at V3, V4, and V5 compared to V2 (*p* = 0.0465, 0.0349, and 0.0349, respectively). Additionally, we observed significantly lower abdominal pain sub scores in the WPI-arm at V8 compared to V2 (*p* = 0.0394). No other post-hoc test, including stool type and frequency (Online Resource 10), reached statistical significance.

### Surrogate markers of health

Original, non-transformed data of all measured biomarkers during the intervention are summarised as median (IQR) in Online Resource 11, including the results of the main statistical analysis (main effects of time, treatment, and BMI, as well as their interactions).

Subsequent post-hoc analyses of the first comparison (PPC vs. PPI) revealed significantly higher serum insulin levels at V6 compared to V2 (*p* = 0.0250) within the PPC-arm. Between the intervention groups, faecal calprotectin levels at V4 were significantly higher in individuals in the PPC-arm compared to PPI (*p* = 0.0340).

When comparing PPI vs. WPI, we observed significantly lower plasma creatinine levels within the lower BMI group (≤ 23.2 kg/m^2^) at V6 compared to V2 (*p* = 0.0230) in the WPI-arm. No other post-hoc test reached statistical significance. The statistical model for the main analysis of the 24-h urine samples failed due missing values and unbalanced data. However, descriptive statistics indicate an increase of the median urea concentration in all arms (Online Resource 11).

## Discussion

In this 3-arm parallel, 4-week dietary intervention study, we have demonstrated that not only the protein source (WPI vs. PPI), but also the presence of a more complex pea protein matrix (PPI vs. PPC) distinctively affects gut microbiota composition and diversity measures in healthy individuals.

Over the course of the intervention, we observed a significant decrease in α-diversity in the arms consuming the PBPs, indicating a reduced within-sample richness and evenness. This effect was modulated by the participants’ BMI, as shown by a significant interaction, whereas β-diversity exhibited a BMI-independent corresponding increase. The association between α-diversity and BMI aligns with previous reports, describing lower microbial diversity with increasing BMI [29, 30]. An increasing α-diversity is generally considered indicative of improved gut health [31], therefore the observed diversity reduction in our study may suggest the onset of an emerging dysbiosis. This observation could potentially be caused by increased proteolytic fermentation and may reflect a selective enrichment of taxa adapted to the dietary substrates entering the colonic microbial ecosystem, suggesting functional adjustment.

At the species level, PPI supplementation resulted in a significant increase in the relative abundance of the lactic acid bacteria (LAB) *Lactobacillus crispatus* and *Limosilactobacillus frumenti*, which is in line with a previously conducted study using the same PPI [18]. Lactic acid bacteria in general are amongst the most used probiotics, due to their range of potential beneficial health effects, including antimicrobial, immunomodulatory, and anti-inflammatory properties [32–34]. Additionally, LAB have been shown to exhibit proteolytic activity in the absence of other energy sources [35]. *Limosilactobacillus frumenti* in particular has been reported to hydrolyse arginine to ammonia to support its growth [36]. When comparing the AA composition of the two protein isolates, PPI contains a higher proportion of arginine than WPI [37], which may explain the observed results. Interestingly, in the WPI-arm, the relative abundance of the AA-fermenting *Acidaminococcus intestini* – a species that was absent in participants in the PPI-arm – decreased significantly [38]. However, these findings may be attributed to the high digestibility of WPI, which likely reduces the amount of protein and AAs reaching the colon for fermentation [39]. According to the 24-h food diaries, protein intake increased equally in both arms (WPI vs. PPI) from 0.9 g to 1.4 g/ kg body mass/ day, which is higher than the recommended daily protein intake of 0.8 to 1.0 g/ kg body mass/ day, therefore reflecting a high protein intake [40]. As the intake did not differ significantly between the arms and as both isolates are considered equally digestible, the differential effect of PPI vs. WPI may be further explained by the presence of other compounds in PPI, including bioactive peptides and polyphenols [41]. This hypothesis is strengthened by the significantly decreased relative abundance of *Eubacterium ventriosum* at V6 compared to V2 in the PPI-arm, as its abundance has been reported to be inversely correlated with polyphenol and flavonoid consumption [42].

In the PPC-arm, we observed a significant increase of the relative abundance of *Anaerobutyricum hallii* over time, also known as *Eubacterium hallii* [43], in addition to an increase in relative abundance of *Bifidobacterium longum* – a well-described species with probiotic characteristics and connections to beneficial health outcomes [44]. *Anaerobutyricum hallii* has been described as a candidate for next-generation probiotics due to its ability to produce butyrate from glucose and lactate as well as propionate from 1,2-propanediol [45]. These substrates are common fermentation products of saccharolytic bacteria, indicating an underlying cross-feeding mechanism [45, 46]. Additionally, it was reported to be involved in detoxifying carcinogens as well as improving glucose homeostasis by increasing the production of gut hormones including GLP-1 and GLP-2 [45, 47, 48]. In both PBP-arms we observed fluctuating relative abundances of *Odoribacter splanchnicus* – a species with multiple proposed probiotic functions [49]. The initial increase and subsequent decrease may reflect adaptive mechanisms within the microbial community in response to substrate availability.

However, although several of the taxa identified in our study have previously been associated with potentially beneficial functions or health outcomes, their increased abundance should not be interpreted as evidence that these effects were functionally expressed in the present study. Furthermore, we did not observe major changes in other clinical or metabolic outcomes, further suggesting that the observed changes in microbial composition did not necessarily translate into measurable physiological benefits. Additionally, while several of the most abundant species were consistently shared across participants, baseline profiling of microbial species also highlights the interindividual variation in gut microbiota composition, emphasising the complexity of assessing supplement-induced changes.

Interestingly, we observed increased fasting insulin levels at the end of the intervention in comparison to baseline in the PPC-arm when investigating changes in surrogate markers of metabolic health. While there is a lack of studies on the effects of high PBP and fibre intake with simultaneously normal fat and carbohydrate on fasting insulin in healthy subjects, this finding contradicts the previously reported insulin-lowering effects of plant-based diets in general [50, 51]. Although time effects for faecal calprotectin were not statistically significant, we observed significantly increased concentrations in the PPC-arm at V4 compared to the PPI-arm. While these findings do not allow conclusions regarding time effects of protein supplementation nor differential effects attributable to the protein source itself, they raise the possibility of a responder phenotype within the population, whereby certain individuals may be more predisposed to gastrointestinal inflammatory responses increasing the upper range of the observed calprotectin concentrations. This hypothesis is consistent with previously reported findings showing increased faecal calprotectin concentrations following incremental increases in PPI supplementation in a subpopulation [18] but requires further validation in a larger cohort. Additionally, increased faecal calprotectin levels have been reported to be associated with microbial dysbiosis, BMI, history of cardiovascular disease and other comorbidities [52], wherefore increased baseline or population levels may have affected study outcomes.

Although indigestion scores increased at V3, V4, and V5 compared with baseline in the PPC-arm, and abdominal pain scores decreased at V8 compared with baseline in the WPI-arm, these changes were observed only within individual intervention arms and were not accompanied by significant between-arm differences. Even though results also indicate changes over time in stool type and frequency, lack of significance in the post-hoc analysis did not allow us to specify the direction of change. Thus, these findings should be interpreted cautiously and do not provide evidence of a consistent intervention-related effect on gastrointestinal symptoms and bowel habits.

Nevertheless, it is important to keep these subtle effects in mind as gut physiology, including transit time, pH, and stool type, has been shown to affect gut microbiota composition [53–55].

### Further strengths and limitations

A strength of this intervention study is the previously mentioned high level of compliance, supported by the food diary results and the returned bags. Both the increased protein intake and the number of returned empty bags did not differ significantly between the study arms, indicating that the different supplements did not differentially influence adherence to the intervention. Data quality prevented proper statistical analysis of urine markers, which would have otherwise been a more objective marker of protein intake compliance. However, the urine urea median increased in all groups indicating that the protein intake did increase in all arms. Furthermore, the implementation of specific dietary inclusion criteria, effectively minimised inter-individual variability in habitual dietary patterns, particularly with regard to dietary fibre and protein intake. The absence of significant differences in participants’ dietary characteristics further underscores the efficacy of these inclusion criteria in achieving a comparable nutritional profile across study groups before the intervention. Nevertheless, although participants were excluded if they used protein or fibre supplements, they were not specifically instructed to exclude pea- or whey-containing foods from their habitual diet. Based on our review of the 24-h food diaries, pea-containing foods did not constitute a significant part of participants’ habitual dietary intake, and consumption patterns remained stable throughout the intervention. We therefore do not expect habitual dietary intake of pea- or whey-derived protein to have meaningfully confounded the observed results.

A key limitation of this study is the use of isolated proteins and a protein concentrate, limiting the generalisability of the findings to whole-food products containing the tested proteins as ingredients. However, we intended to employ products with lower matrix complexity allowing for clearer attribution of the observed effects to the supplements themselves and minimising confounding influences from other nutrients, vitamins, and minerals. Nevertheless, differences between the study arms may still have been partly attributable to differences in food matrix characteristics beyond protein, dietary fibre, carbohydrate, and fat content, including the degree of processing and the presence of other bioactive compounds. Additionally, the protein supplementation affected the overall macronutrient composition over time, which needs to be taken into consideration when interpreting the results.

Even though we anticipated that participants would maintain an isocaloric diet throughout the intervention, we observed significant differences over time for body mass and BMI. Although post-hoc analyses did not detect significant changes in energy intake, the increase in body mass and BMI in the PPI-arm suggests that daily calorie intake may have increased for some individuals. However, even though the median body mass change from 22.8 to 23.4 kg reached statistical significance, it may not be physiologically relevant. As the majority of participants were females – whose body mass naturally fluctuates throughout the menstrual cycle – these variations may partially explain the observed increase [56]. The predominance of females, and the inclusion of healthy, mostly younger adults, may further limit the transferability of the observations to broader populations. The included stratification by baseline BMI was exploratory in nature and was based on previously published findings reporting associations between certain microbial species and BMI as well as differential effects among normal-weight, overweight, and obese populations with regard to microbiota diversity and related outcomes [26, 27]. While BMI significantly increased in the PPI-arm over the intervention, it has to be noted that it only increased slightly (22.8 kg/m^2^ to 23.4 kg/m^2^) exceeding the cut-off for the grouping by only 0.2 kg/m^2^. This is important to highlight, even though we do not expect that this increase has substantially influenced our results and may be attributable to physiological weight fluctuation. Additionally, our classification deviates from the commonly used thresholds for normal weight and overweight (23.2 kg/m^2^ vs. 24.9 kg/m^2^ [57]. However, we chose to group participants based on the median as this approach resulted in the most balanced group sizes possible. Nevertheless, the number of participants within the BMI subgroups were relatively small and remained unequal, which limits the statistical power of these exploratory analyses. This limitation of a small sample size also applies to the study overall, as the intervention was completed with fewer participants than initially anticipated due to logistical constrains.

Due to the filtering approach applied when analysing changes at the species level, which focused on significant time:treatment and time:treatment:BMI interactions, we limited the number of multiple comparisons and prioritised findings that could be meaningfully attributed to both intervention and time effects. Consequently, other main effects, including time, treatment, or BMI effects, were not reported, which is an important consideration when interpreting the results.

Furthermore, the parallel design of the study limits the extent to which observed treatment effects can be attributed solely to the intervention, and it is important to consider inter-individual differences between the study populations that may have affected the reported results. Additionally, the absence of a control arm prevents us from assessing whether the observed changes occurred due to adaptation to the collection of biological samples and questionnaires. However, we believe that the 2-week baseline period before the start of the intervention likely mitigated this risk by providing participants with the opportunity to get acquainted with the study procedures.

## Conclusion

In this 3-arm, 4-week dietary intervention study, we directly compared the effect of protein source (PPI vs. WPI) and matrix complexity (PPC vs. PPI) on gut microbiota composition in healthy adults. The intake of both PBPs was associated with a BMI-modulated reduction in α-diversity, alongside BMI-independent shifts in β-diversity. Additionally, we observed distinct relative abundance changes at the species level, indicating matrix-specific effects. While the supplementation of PPI resulted in an increased relative abundance of *Lactobacillus crispatus* and *Limosilactobacillus frumenti*, supplementation of PPC increased relative abundances of *Anaerobutyricum hallii* and *Bifidobacterium longum*.

To further elucidate the functional consequences of these microbial shifts, additional studies with a larger study population are warranted to enable subgroup analyses and to explore underlying metabolic pathways within a precision nutrition framework.

## Supplementary Information

Supplementary Material 1. Online Resource 1: Inclusion and exclusion criteria. Online Resource 2: Recruitment flow diagram. Online Resource 3: Gut microbiota diversity measures. Online Resource 4: Most abundant species. Online Resource 5: Relative abundance data of species with significant interactions (PPC vs. PPI). Online Resource 6: Relative abundance data of species with significant interactions (PPI vs. WPI). Online Resource 7: Anthropometric measures and dietary intake. Online Resource 8: Physical activity. Online Resource 9: Gastrointestinal symptom rating scale scoresOnline Resource 10: Bristol stool scale scores. Online Resource 11: Biomarkers in blood, urine and faecal samples.

Supplementary Material 2: Inclusion and exclusion criteria

Supplementary Material 3: Recruitment flow diagram

Supplementary Material 4: Gut microbiota diversity measures

Supplementary Material 5: Most abundant species

Supplementary Material 6: Relative abundance data of species with significant interactions (PPC vs. PPI)

Supplementary Material 7: Relative abundance data of species with significant interactions (PPI vs. WPI)

Supplementary Material 8: Anthropometric measures and dietary intake

Supplementary Material 9: Physical activity

Supplementary Material 10: Gastrointestinal symptom rating scale scores

Supplementary Material 11: Bristol stool scale scores

Supplementary Material 12: Biomarkers in blood, urine and faecal samples

## Data Availability

The datasets presented in this article are not readily available because restrictions apply to the availability of some, or all data generated or analysed during this study to preserve patient confidentiality or because they were used under license. The corresponding author will on request detail the restrictions and any conditions under which access to some data may be provided. Requests to access the datasets should be directed to forskningsdata@oru.se.
