# Supplementary material for: Differential effects of pea protein isolate, pea protein concentrate, and whey protein isolate supplementation on gut microbiota and metabolic markers in healthy adults: a 3-arm parallel randomised intervention
Source: Eur J Nutr. 2026 Nov 11;65(8):299. doi: 10.1007/s00394-026-04143-z (PMC13755021; doi:10.1007/s00394-026-04143-z)
Supplement: Supplementary file 1 [file 394_2026_4143_MOESM10_ESM.docx]

**Differential effects of pea protein isolate, pea protein concentrate, and whey protein isolate supplementation on gut microbiota and metabolic markers in healthy adults: a 3-arm parallel randomised intervention**

Annalena Kamm^a,b^, Samira Prado^a,b,c^, Katharina Dannenberg^a,d^, Dirk Repsilber^a,b^, Tatiana M. Marques^a,b^, Robert J. Brummer^a,b^

^a^School of Medical Sciences, Faculty of Medicine and Health, Örebro University, Örebro, Sweden

^b^Food and Health Centre, Örebro University, Örebro, Sweden

^c^School of Science and Technology, Faculty of Business, Science and Engineering, Örebro University, Sweden

^d^Clinical Genomics Örebro, Science for Life Laboratory, Örebro University, Örebro, Sweden

**Corresponding Authors**

Annalena Kamm

annalena.kamm@oru.se

ORCiD: 0009-0002-9389-4172

Samira Prado

samira.prado@oru.se

ORCiD: 0000-0003-4627-6291

**Online Resource 9** Gastrointestinal symptom rating scale scores during the whole study period evaluated using the Gastrointestinal Symptom Rating Scale

|  | Week | PPC (n=19) | PPI (n=19) | WPI (n=18) | p-value | | | | | |
| --- | --- | --- | --- | --- | --- | --- | --- | --- | --- | --- |
|  |  | *Median (IQR)* | | | *PPC vs. PPI* | | | *PPI vs. WPI* | | |
|  |  |  |  |  | *T* | *TR* | *T-TR* | *T* | *TR* | *T-TR* |
| Reflux Score | 1 | 1.0 (1.0–1.0) | 1.0 (1.0–1.0) | 1.0 (1.0–1.0) | **0.0326** | 0.4775 | **0.0336** | 0.8994 | 0.7981 | 0.2539 |
|  | 2 | 1.0 (1.0–1.0) | 1.0 (1.0–1.2) | 1.0 (1.0–1.0) |  |  |  |  |  |  |
|  | 3 | 1.0 (1.0–1.8) | 1.0 (1.0–1.0) | 1.0 (1.0–1.4) |  |  |  |  |  |  |
|  | 4 | 1.0 (1.0–1.0) | 1.0 (1.0–1.0) | 1.0 (1.0–1.0) |  |  |  |  |  |  |
|  | 5 | 1.0 (1.0–1.5) | 1.0 (1.0–1.0) | 1.0 (1.0–1.0) |  |  |  |  |  |  |
|  | 6 | 1.0 (1.0–1.5) | 1.0 (1.0–1.0) | 1.0 (1.0–1.0) |  |  |  |  |  |  |
|  | 7 | 1.0 (1.0–1.0) | 1.0 (1.0–1.0) | 1.0 (1.0–1.0) |  |  |  |  |  |  |
|  | 8 | 1.0 (1.0–1.0) | 1.0 (1.0–1.2) | 1.0 (1.0–1.0) |  |  |  |  |  |  |
| Abdominal Pain Score | 1 | 1.3 (1.3–2.0) | 1.3 (1.0–2.0) | 1.3 (1.3–1.9) | **0.0056** | 0.3932 | 0.4729 | **0.0354** | 0.5858 | 0.2694 |
|  | 2 | 1.3 (1.3–1.7) | 1.3 (1.0–1.7) | 1.7 (1.0–2.0) |  |  |  |  |  |  |
|  | 3 | 1.7 (1.0–2.2) | 2.0 (1.0–2.0) | 1.3 (1.0–1.9) |  |  |  |  |  |  |
|  | 4 | 1.7 (1.2–2.3) | 1.7 (1.2–1.9) | 1.5 (1.0–2.2) |  |  |  |  |  |  |
|  | 5 | 1.7 (1.0–2.0) | 1.3 (1.0–1.7) | 1.3 (1.1–2.2) |  |  |  |  |  |  |
|  | 6 | 1.7 (1.0–2.2) | 1.0 (1.0–1.5) | 1.3 (1.0–2.2) |  |  |  |  |  |  |
|  | 7 | 1.3 (1.0–1.7) | 1.3 (1.0–1.7) | 1.3 (1.1–1.9) |  |  |  |  |  |  |
|  | 8 | 1.3 (1.0–1.7) | 1.0 (1.0–1.3) | 1.0 (1.0–1.7) |  |  |  |  |  |  |
| Indigestion Score | 1 | 2.0 (1.8–2.6) | 2.2 (1.2–2.5) | 2.1 (1.8–2.7) | **< 0.0001** | 0.3336 | 0.7038 | **0.0012** | 0.7293 | 0.5887 |
|  | 2 | 2.0 (1.5–2.4) | 1.8 (1.4–2.1) | 1.8 (1.3–2.2) |  |  |  |  |  |  |
|  | 3 | 2.2 (1.8–2.9) | 2.2 (1.8–2.8) | 2.2 (1.6–2.5) |  |  |  |  |  |  |
|  | 4 | 2.5 (1.9–3.1) | 2.0 (1.4–2.8) | 2.5 (1.8–2.7) |  |  |  |  |  |  |
|  | 5 | 2.5 (1.6–3.2) | 2.0 (1.6–2.4) | 2.0 (1.6–2.7) |  |  |  |  |  |  |
|  | 6 | 2.5 (1.8–3.5) | 2.0 (1.8–2.6) | 1.9 (1.5–2.8) |  |  |  |  |  |  |
|  | 7 | 1.8 (1.5–2.4) | 1.5 (1.5–2.1) | 1.8 (1.5–2.6) |  |  |  |  |  |  |
|  | 8 | 2.0 (1.5–2.1) | 1.8 (1.4–2.2) | 1.6 (1.5–2.2) |  |  |  |  |  |  |
| Diarrhoea Score | 1 | 1.3 (1.0–2.3) | 1.0 (1.0–1.3) | 1.3 (1.0–1.6) | 0.5455 | 0.4235 | 0.7820 | 0.7369 | 0.6887 | 0.0967 |
|  | 2 | 1.3 (1.0–1.7) | 1.0 (1.0–2.0) | 1.0 (1.0–1.7) |  |  |  |  |  |  |
|  | 3 | 1.3 (1.0–2.0) | 1.3 (1.0–2.5) | 1.2 (.01–2.2) |  |  |  |  |  |  |
|  | 4 | 1.3 (1.0–1.7) | 1.3 (1.0–1.3) | 1.2 (1.0–1.3) |  |  |  |  |  |  |
|  | 5 | 1.3 (1.0–1.8) | 1.3 (1.0–1.7) | 1.0 (1.0–1.3) |  |  |  |  |  |  |
|  | 6 | 1.3 (1.0–2.0) | 1.0 (1.0–1.3) | 1.5 (1.0–1.7) |  |  |  |  |  |  |
|  | 7 | 1.3 (1.0–1.5) | 1.0 (1.0–1.3) | 1.3 (1.0–2.2) |  |  |  |  |  |  |
|  | 8 | 1.0 (1.0–1.8) | 1.0 (1.0–1.4) | 1.0 (1.0–1.7) |  |  |  |  |  |  |
| Constipation Score | 1 | 1.0 (1.0–1.9) | 1.3 (1.0–2.8) | 1.2 (1.0–1.6) | 0.0593 | 0.3727 | 0.3957 | 0.4610 | 0.7216 | 0.3277 |
|  | 2 | 1.7 (1.0–1.7) | 1.3 (1.0–2.7) | 1.3 (1.0–2.0) |  |  |  |  |  |  |
|  | 3 | 1.7 (1.3–2.2) | 2.0 (1.0–3.0) | 1.7 (1.0–2.3) |  |  |  |  |  |  |
|  | 4 | 1.7 (1.0–2.0) | 1.3 (1.0–2.7) | 1.5 (1.0–2.3) |  |  |  |  |  |  |
|  | 5 | 1.7 (1.0–2.7) | 1.7 (1.0–3.0) | 1.3 (1.0–3.2) |  |  |  |  |  |  |
|  | 6 | 1.7 (1.2–2.3) | 1.7 (1.0–2.5) | 1.7 (1.0–2.8) |  |  |  |  |  |  |
|  | 7 | 1.0 (1.0–1.5) | 1.3 (1.0–2.7) | 1.8 (1.3–2.0) |  |  |  |  |  |  |
|  | 8 | 1.3 (1.0–1.7) | 1.3 (1.0–2.8) | 1.7 (1.0–2.2) |  |  |  |  |  |  |
| Total GSRS Score | 1 | 1.6 (1.3–2.0) | 1.5 (1.4–1.9) | 1.6 (1.3–1.8) | **< 0.0001** | 0.5870 | 0.3510 | **0.0298** | 0.8764 | 0.4718 |
|  | 2 | 1.7 (1.4–1.8) | 1.5 (1.3–2.0) | 1.5 (1.3–1.7) |  |  |  |  |  |  |
|  | 3 | 1.7 (1.4–2.4) | 1.7 (1.5–2.1) | 1.8 (1.4–2.0) |  |  |  |  |  |  |
|  | 4 | 1.9 (1.6–2.1) | 1.6 (1.3–2.0) | 1.6 (1.4–2.2) |  |  |  |  |  |  |
|  | 5 | 1.7 (1.5–2.4) | 1.7 (1.3–2.0) | 1.5 (1.3–2.5) |  |  |  |  |  |  |
|  | 6 | 2.0 (1.6–2.3) | 1.5 (1.3–1.9) | 1.6 (1.4–2.2) |  |  |  |  |  |  |
|  | 7 | 1.4 (1.2–1.9) | 1.5 (1.2–1.9) | 1.6 (1.4–2.1) |  |  |  |  |  |  |
|  | 8 | 1.4 (1.3–1.8) | 1.4 (1.2–1.9) | 1.5 (1.2–1.8) |  |  |  |  |  |  |

GSRS: Gastrointestinal Symptom Rating Scale, IQR: Interquartile Range, PPC: Pea Protein Concentrate, PPI: Pea Protein Isolate, T: Time, TR: Treatment, T-TR: Time-Treatment Interaction, WPI: Whey Protein Isolate; GSRS scores range from 1, representing no discomfort to 7, representing very severe discomfort; Significances are indicated in bold (descriptive p-values originating from a non-parametric factorial repeated-measures model with the factors time, treatment, and their interactions), α = 0.05
