# Supplementary material for: Differential effects of pea protein isolate, pea protein concentrate, and whey protein isolate supplementation on gut microbiota and metabolic markers in healthy adults: a 3-arm parallel randomised intervention
Source: Eur J Nutr. 2026 Nov 11;65(8):299. doi: 10.1007/s00394-026-04143-z (PMC13755021; doi:10.1007/s00394-026-04143-z)
Supplement: Supplementary file 2 [file 394_2026_4143_MOESM11_ESM.docx]

**Differential effects of pea protein isolate, pea protein concentrate, and whey protein isolate supplementation on gut microbiota and metabolic markers in healthy adults: a 3-arm parallel randomised intervention**

Annalena Kamm^a,b^, Samira Prado^a,b,c^, Katharina Dannenberg^a,d^, Dirk Repsilber^a,b^, Tatiana M. Marques^a,b^, Robert J. Brummer^a,b^

^a^School of Medical Sciences, Faculty of Medicine and Health, Örebro University, Örebro, Sweden

^b^Food and Health Centre, Örebro University, Örebro, Sweden

^c^School of Science and Technology, Faculty of Business, Science and Engineering, Örebro University, Sweden

^d^Clinical Genomics Örebro, Science for Life Laboratory, Örebro University, Örebro, Sweden

**Corresponding Authors**

Annalena Kamm

annalena.kamm@oru.se

ORCiD: 0009-0002-9389-4172

Samira Prado

samira.prado@oru.se

ORCiD: 0000-0003-4627-6291

**Online Resource 10** Bristol stool scale scores during the whole study period

|  | Week | PPC (n=19) | PPI (n=19) | WPI (n=18) | p-value | | | | | |
| --- | --- | --- | --- | --- | --- | --- | --- | --- | --- | --- |
|  |  | *Median (IQR)* | | | *PPC vs. PPI* | | | *PPI vs. WPI* | | |
|  |  |  |  |  | *T* | *TR* | *T-TR* | *T* | *TR* | *T-TR* |
| Average Stool Type | 1 | 3.8 (3.4–4.4) | 3.7 (3.1–4.1) | 3.6 (3.1–4.2) | 0.9171 | 0.5148 | 0.1326 | **0.0477** | 0.4450 | 0.7207 |
|  | 2 | 3.6 (3.2–3.9) | 4.1 (3.7–4.8) | 3.7 (3.2–4.4) |  |  |  |  |  |  |
|  | 3 | 3.7 (3.4–4.4) | 3.9 (3.7–4.6) | 4.0 (3.2–4.3) |  |  |  |  |  |  |
|  | 4 | 3.6 (3.3–3.8) | 3.8 (3.2–4.6) | 3.6 (2.8–4.2) |  |  |  |  |  |  |
|  | 5 | 3.8 (3.2–4.2) | 3.9 (3.0–4.4) | 3.7 (2.9–4.0) |  |  |  |  |  |  |
|  | 6 | 4.0 (3.0–4.0) | 3.7 (3.1–4.5) | 3.3 (2.8–4.1) |  |  |  |  |  |  |
|  | 7 | 3.9 (3.4–4.3) | 3.6 (3.0–4.9) | 3.8 (3.1–4.4) |  |  |  |  |  |  |
|  | 8 | 3.8 (3.3–4.2) | 3.3 (2.9–4.7) | 3.5 (3.0–4.0) |  |  |  |  |  |  |
| Average Frequency/day | 1 | 1.0 (0.7–1.1) | 1.0 (1.0–1.2) | 1.0 (0.7–1.1) | 0.1102 | 0.2381 | 0.4633 | **0.0292** | **0.0286** | 0.7409 |
|  | 2 | 1.0 (0.7–1.5) | 1.2 (1–1.5) | 1.0 (0.8–1.2) |  |  |  |  |  |  |
|  | 3 | 1.1 (0.9–1.4) | 1.1 (1.0–1.3) | 1.0 (0.8–1.3) |  |  |  |  |  |  |
|  | 4 | 1.0 (0.8–1.4) | 1.2 (1.1–1.6) | 1.1 (0.9–1.4) |  |  |  |  |  |  |
|  | 5 | 0.9 (0.7–1.3) | 1.1 (1.0–1.6) | 1.2 (0.7–1.4) |  |  |  |  |  |  |
|  | 6 | 1.0 (0.8–1.2) | 1.3 (1–1.7) | 1.0 (0.8–1.1) |  |  |  |  |  |  |
|  | 7 | 1.0 (0.7–1.3) | 1.1 (0.9–1.5) | 0.9 (0.9–1.1) |  |  |  |  |  |  |
|  | 8 | 0.9 (0.7–1.3) | 1.1 (0.8–1.6) | 0.9 (0.7–1.1) |  |  |  |  |  |  |

IQR: Interquartile Range, PPC: Pea Protein Concentrate, PPI: Pea Protein Isolate, T: Time, TR: Treatment, T-TR: Time-Treatment Interaction, WPI: Whey Protein Isolate; Significances are indicated in bold (descriptive p-values originating from a non-parametric factorial repeated-measures model with the factors time, treatment, and their interactions), α = 0.05
