# Supplementary material for: Differential effects of pea protein isolate, pea protein concentrate, and whey protein isolate supplementation on gut microbiota and metabolic markers in healthy adults: a 3-arm parallel randomised intervention
Source: Eur J Nutr. 2026 Nov 11;65(8):299. doi: 10.1007/s00394-026-04143-z (PMC13755021; doi:10.1007/s00394-026-04143-z)
Supplement: Supplementary file 4 [file 394_2026_4143_MOESM1_ESM.zip › Online Resource 11_Biomarkers in blood, urine and faecal samples_revision.docx]

**Differential effects of pea protein isolate, pea protein concentrate, and whey protein isolate supplementation on gut microbiota and metabolic markers in healthy adults: a 3-arm parallel randomised intervention**

Annalena Kamm^a,b^, Samira Prado^a,b,c^, Katharina Dannenberg^a,d^, Dirk Repsilber^a,b^, Tatiana M. Marques^a,b^, Robert J. Brummer^a,b^

^a^School of Medical Sciences, Faculty of Medicine and Health, Örebro University, Örebro, Sweden

^b^Food and Health Centre, Örebro University, Örebro, Sweden

^c^School of Science and Technology, Faculty of Business, Science and Engineering, Örebro University, Sweden

^d^Clinical Genomics Örebro, Science for Life Laboratory, Örebro University, Örebro, Sweden

**Corresponding Authors**

Annalena Kamm

annalena.kamm@oru.se

ORCiD: 0009-0002-9389-4172

Samira Prado

samira.prado@oru.se

ORCiD: 0000-0003-4627-6291

**Online Resource 11** Biomarkers in blood, urine and faecal samples during the intervention

|  | | Visit | PPC | | PPI | | WPI | |
| --- | --- | --- | --- | --- | --- | --- | --- | --- |
|  | |  | *Median (IQR)* | *n* | *Median (IQR)* | *n* | *Median (IQR)* | *n* |
| Fasting Blood Samples | | | | | | | | |
| CRP  (mg/L) | | 2 | 0.5 (0.3–1.7) | 17 | 0.4 (0.3–1.0) | 17 | 0.3 (0.2–0.6) | 16 |
|  |  | 4 | 0.6 (0.3–1.5) | 19 | 0.5 (0.3–0.6) | 17 | 0.3 (0.2–0.7) | 17 |
|  |  | 6 | 0.7 (0.4–1.9) | 16 | 0.5 (0.3–0.7) | 19 | 0.5 (0.3–0.8) | 13 |
| Factor | | ***T*** | ***TR*** | ***BMI*** | ***T-BMI*** | ***T-TR*** | ***TR-BMI*** | ***T-TR-BMI*** |
| p-value | *PPC vs. PPI PPI vs. WPI* | 0.5746 0.1860 | 0.4090 0.5754 | 0.1637 0.3048 | 0.8619 0.9874 | 0.2570 0.8605 | 0.4769 0.9070 | 0.6195 0.5533 |
| Glucose  (mmol/L) | | 2 | 4.9 (4.7–5.2) | 17 | 5.1 (4.9–5.5) | 19 | 5.1 (4.8–5.2) | 16 |
|  |  | 4 | 5.0 (4.9–5.3) | 19 | 4.9 (4.8–5.4) | 17 | 4.9 (4.7–5.2) | 17 |
|  |  | 6 | 5.0 (4.8–5.2) | 17 | 5.0 (4.7–5.3) | 19 | 4.9 (4.7–5.1) | 13 |
| Factor | | ***T*** | ***TR*** | ***BMI*** | ***T-BMI*** | ***T-TR*** | ***TR-BMI*** | ***T-TR-BMI*** |
| p-value | *PPC vs. PPI PPI vs. WPI* | 0.7002 0.4280 | 0.4308 0.9371 | 0.5691 0.4444 | 0.9725 0.1122 | 0.1479 0.4835 | 0.2686 0.2389 | 0.8356 0.2589 |
| Insulin  (mIU/L) | | 2 | 4.7 (3.2–9.1) | 17 | 5.2 (3.4–9.2) | 19 | 4.9 (2.6–7.5) | 17 |
|  |  | 4 | 6.9 (5.2–11.2) | 19 | 5.0 (3.4–8.0) | 17 | 5.3 (3.7–7.3) | 17 |
|  |  | 6 | 5.8 (4.4–9.5) | 17 | 7.2 (6.0–10.7) | 19 | 6.0 (3.2–9.2) | 14 |
| Factor | | ***T*** | ***TR*** | ***BMI*** | ***T-BMI*** | ***T-TR*** | ***TR-BMI*** | ***T-TR-BMI*** |
| p-value | *PPC vs. PPI PPI vs. WPI* | **0.0264** 0.0703 | 0.5624 0.4928 | 0.2579 0.1933 | 0.4793 0.6350 | 0.1652 0.5956 | 0.2373 0.2021 | 0.4285 0.4194 |
| Total Cholesterol (mmol/L) | | 2 | 4.4 (3.9–4.7) | 18 | 4.2 (3.9–5.1) | 19 | 4.5 (3.9–4.9) | 16 |
|  |  | 4 | 4.2 (3.6–4.7) | 19 | 4.4 (3.9–4.6) | 17 | 4.4 (4.2–4.9) | 17 |
|  |  | 6 | 4.0 (3.4–4.4) | 16 | 4.2 (3.7–4.4) | 19 | 4.0 (3.4–4.8) | 12 |
| *Factor* | | ***T*** | ***TR*** | ***BMI*** | ***T-BMI*** | ***T-TR*** | ***TR-BMI*** | ***T-TR-BMI*** |
| p-value | *PPC vs. PPI PPI vs. WPI* | **0.0253** 0.0502 | 0.5712 0.9167 | 0.4074 0.2185 | 0.4464 0.0756 | 0.2584 0.8407 | 0.4375 0.7756 | 0.6350 0.1048 |
| HDL Cholesterol (mmol/L) | | 2 | 1.4 (1.3–1.5) | 18 | 1.7 (1.4–2.0) | 19 | 1.6 (1.5–1.9) | 16 |
|  |  | 4 | 1.5 (1.4–1.6) | 19 | 1.7 (1.3–2.0) | 17 | 1.6 (1.4–1.8) | 17 |
|  |  | 6 | 1.4 (1.2–1.6) | 16 | 1.6 (1.4–1.9) | 19 | 1.6 (1.3–1.8) | 12 |
| *Factor* | | ***T*** | ***TR*** | ***BMI*** | ***T-BMI*** | ***T-TR*** | ***TR-BMI*** | ***T-TR-BMI*** |
| p-value | *PPC vs. PPI PPI vs. WPI* | 0.7830 0.5758 | 0.3653 0.9329 | **0.0356** 0.0822 | 0.5573 0.0963 | 0.1243 0.6805 | 0.7292 0.6423 | 0.7848 0.4552 |
| LDL Cholesterol (mmol/L) | | 2 | 2.8 (2.4–3.0) | 18 | 2.6 (2.3–3.2) | 19 | 2.5 (2.3–3.2) | 16 |
|  |  | 4 | 2.8 (1.9–3.2) | 19 | 2.6 (2.2–3.0) | 17 | 2.7 (2.3–3.0) | 17 |
|  |  | 6 | 2.7 (1.7–2.7) | 16 | 2.7 (2.3–2.8) | 19 | 2.3 (1.8–2.9) | 13 |
| *Factor* | | ***T*** | ***TR*** | ***BMI*** | ***T-BMI*** | ***T-TR*** | ***TR-BMI*** | ***T-TR-BMI*** |
| p-value | *PPC vs. PPI PPI vs. WPI* | 0.0914 0.2199 | 0.6178 0.9051 | 0.7273 0.9867 | 0.1196 **0.0271** | 0.1535 0.7299 | 0.6588 0.7132 | 0.2142 0.1341 |
| Triglycerides  (mmol/L) | | 2 | 0.8 (0.6–0.9) | 17 | 0.7 (0.6–0.8) | 18 | 0.8 (0.6–0.9) | 16 |
|  |  | 4 | 0.6 (0.6–1.0) | 18 | 0.7 (0.6–0.8) | 17 | 0.7 (0.6–1.0) | 17 |
|  |  | 6 | 0.6 (0.6–0.8) | 16 | 0.6 (0.5–0.8) | 19 | 0.7 (0.5–0.9) | 13 |
| *Factor* | | ***T*** | ***TR*** | ***BMI*** | ***T-BMI*** | ***T-TR*** | ***TR-BMI*** | ***T-TR-BMI*** |
| p-value | *PPC vs. PPI PPI vs. WPI* | 0.0914 0.2199 | 0.6178 0.9051 | 0.7273 0.9867 | 0.1196 **0.0271** | 0.1535 0.7299 | 0.6588 0.7132 | 0.2142 0.1341 |
| Urea  (mmol/L) | | 2 | 3.9 (3.7–4.8) | 18 | 4.4 (3.5–5.3) | 19 | 4.0 (3.7–4.5) | 16 |
|  |  | 4 | 4.7 (3.9–5.5) | 19 | 5.0 (3.6–5.6) | 17 | 4.7 (4.0–5.5) | 17 |
|  |  | 6 | 4.7 (3.9–5.2) | 16 | 4.3 (3.8–5.9) | 19 | 4.6 (3.8–5.3) | 12 |
| *Factor* | | ***T*** | ***TR*** | ***BMI*** | ***T-BMI*** | ***T-TR*** | ***TR-BMI*** | ***T-TR-BMI*** |
| p-value | *PPC vs. PPI PPI vs. WPI* | 0.0084 0.1298 | 0.5458 0.1200 | 0.4438 0.0712 | 0.2401 **0.0182** | 0.5035 0.9289 | 0.3799 0.9536 | 0.1248 0.3682 |
| Creatinine  (µmol/L) | | 2 | 68.5  (60.0–79.3) | 18 | 61.0  (57.5–68.5) | 19 | 60.5  (53.3–72.8) | 16 |
|  |  | 4 | 72.0  (61.5–75.0) | 19 | 61.0  (55.0–71.0) | 17 | 62.0  (53.0–71.0) | 17 |
|  |  | 6 | 64.5  (58.0–73.3) | 16 | 58.0  (50.0–65.5) | 19 | 54.0  (47.0–63.0) | 13 |
| *Factor* | | ***T*** | ***TR*** | ***BMI*** | ***T-BMI*** | ***T-TR*** | ***TR-BMI*** | ***T-TR-BMI*** |
| p-value | *PPC vs. PPI PPI vs. WPI* | **0.0301 0.0276** | 0.3595 0.3993 | 0.0638 0.7232 | 0.2000 **0.0158** | 0.6502 0.8833 | 0.4821 0.5804 | 0.4673 0.6770 |
| 24-h Urine Samples | | | | | | | | |
| Urea  (mmol/day) | | 2 | 319.0  (253.0–329.0) | 9 | 269.0  (208.0–332.5) | 11 | 211.5  (134.8–351.0) | 10 |
|  |  | 4 | 347.0  (307.0–360.0) | 5 | 379.0  (283.8–509.8) | 12 | 345.0  (267.0–500.0) | 11 |
|  |  | 6 | 429.0  (383.5–466.5) | 7 | 440.0  (344.0–705.8) | 10 | 461.0  (386.0–537.3) | 8 |
| *Factor* | | ***T*** | ***TR*** | ***BMI*** | ***T-BMI*** | ***T-TR*** | ***TR-BMI*** | ***T-TR-BMI*** |
| p-value | *PPC vs. PPI PPI vs. WPI* | -  **0.0430** | -  0.4977 | -  - | -  - | -  0.7655 | -  - | -  - |
| Protein intake calculated from urine urea  (g/kg body mass/day) | | 2 | 1.0  (0.8–1.0) | 9 | 1.0  (0.8–1.1) | 11 | 0.7  (0.6–0.9) | 10 |
|  |  | 4 | 1.1  (1.1–1.1) | 5 | 1.2  (1.1–1.4) | 12 | 1.2  (1.0–1.5) | 11 |
|  |  | 6 | 1.2  (1.1–1.4) | 7 | 1.4  (1.2–1.9) | 10 | 1.3  (1.2–1.4) | 8 |
| *Factor* | | ***T*** | ***TR*** | ***BMI*** | ***T-BMI*** | ***T-TR*** | ***TR-BMI*** | ***T-TR-BMI*** |
| p-value | *PPC vs. PPI PPI vs. WPI* | -  0.0196 | -  **0.2099** | -  - | -  - | -  0.7570 | -  - | -  - |
| Uric acid  (mmol/day) | | 2 | 3.0  (2.8–3.4) | 9 | 2.7  (2.4–3.0) | 11 | 2.5  (2.1–3.1) | 10 |
|  |  | 4 | 3.6  (3.4–4.4) | 5 | 3.3  (2.6–3.9) | 12 | 2.6  (2.5–3.2) | 11 |
|  |  | 6 | 3.7  (3.6–4.0) | 7 | 3.5  (2.8–5.1) | 10 | 2.7  (2.6–3.6) | 8 |
| *Factor* | | ***T*** | ***TR*** | ***BMI*** | ***T-BMI*** | ***T-TR*** | ***TR-BMI*** | ***T-TR-BMI*** |
| *p-value* | *PPC vs. PPI PPI vs. WPI* | -  0.1285 | -  0.4403 | -  - | -  - | -  0.5003 | -  - | -  - |
| Creatinine  (mmol/day) | | 2 | 13.4  (7.9–14.3) | 9 | 10.0  (9.6–12.2) | 11 | 8.3  (5.4–12.0) | 10 |
|  |  | 4 | 9.8  (9.7–11.9) | 5 | 9.9  (7.6–10.9) | 12 | 10.4  (8.5–14.0) | 11 |
|  |  | 6 | 11.0  (10.8–13.2) | 7 | 10.9  (9.2–13.3) | 10 | 10.7  (9.0–11.7) | 8 |
| *Factor* | | ***T*** | ***TR*** | ***BMI*** | ***T-BMI*** | ***T-TR*** | ***TR-BMI*** | ***T-TR-BMI*** |
| p-value | *PPC vs. PPI PPI vs. WPI* | -  0.0668 | -  0.8824 | -  - | -  - | -  0.9241 | -  - | -  - |
| Faecal Samples | | | | | | | | |
| Calprotectin  (mg/kg) | | 2 | 10.0  (10.0–38.8) | 16 | 10.0  (10.0–45.0) | 18 | 10.0  (10.0–30.2) | 17 |
|  |  | 4 | 31.0  (10.0–103.8) | 17 | 10.0  (10.0–20.0) | 18 | 10.0  (10.0–30.0) | 17 |
|  |  | 6 | 10.0  (10.0–84.5) | 18 | 10.0  (10.0–10.0) | 19 | 10.0  (10.0–28.0) | 18 |
| *Factor* | | ***T*** | ***TR*** | ***BMI*** | ***T-BMI*** | ***T-TR*** | ***TR-BMI*** | ***T-TR-BMI*** |
| p-value | *PPC vs. PPI PPI vs. WPI* | 0.8468  0.7250 | **0.0377**  0.3736 | 0.3489  0.9762 | 0.7309  0.3989 | 0.3393  0.7743 | 0.9914  0.2926 | 0.1357  0.6136 |

CRP: C-reactive Protein, HDL: High-Density Lipoprotein, IQR: Interquartile Range, LDL: Low-Density Lipoprotein, PPC: Pea Protein Concentrate, PPI: Pea Protein Isolate, T: Time, T-BMI: Time-BMI Interaction, T-TR: Time-Treatment Interaction, TR: Treatment, TR-BMI: Treatment-BMI Interaction, T-TR-BMI: Time-Treatment-BMI Interaction, WPI: Whey Protein Isolate; Significances are indicated in bold (descriptive p-values originating from a non-parametric factorial repeated-measures model with the factors time, treatment, BMI, and their interactions), α = 0.05
