# Supplementary material for: Differential effects of pea protein isolate, pea protein concentrate, and whey protein isolate supplementation on gut microbiota and metabolic markers in healthy adults: a 3-arm parallel randomised intervention
Source: Eur J Nutr. 2026 Nov 11;65(8):299. doi: 10.1007/s00394-026-04143-z (PMC13755021; doi:10.1007/s00394-026-04143-z)
Supplement: Supplementary file 4 [file 394_2026_4143_MOESM1_ESM.zip › Online Resource 1_Inclusion and exclusion criteria_revision.docx]

**Differential effects of pea protein isolate, pea protein concentrate, and whey protein isolate supplementation on gut microbiota and metabolic markers in healthy adults: a 3-arm parallel randomised intervention**

Annalena Kamm^a,b^, Samira Prado^a,b,c^, Katharina Dannenberg^a,d^, Dirk Repsilber^a,b^, Tatiana M. Marques^a,b^, Robert J. Brummer^a,b^

^a^School of Medical Sciences, Faculty of Medicine and Health, Örebro University, Örebro, Sweden

^b^Food and Health Centre, Örebro University, Örebro, Sweden

^c^School of Science and Technology, Faculty of Business, Science and Engineering, Örebro University, Sweden

^d^Clinical Genomics Örebro, Science for Life Laboratory, Örebro University, Örebro, Sweden

**Corresponding Authors**

Annalena Kamm

annalena.kamm@oru.se

ORCiD: 0009-0002-9389-4172

Samira Prado

samira.prado@oru.se

ORCiD: 0000-0003-4627-6291

|  | **Inclusion Criteria** | **Exclusion Criteria** |
| --- | --- | --- |
| **General Characteristics** | Age:18–45 years; BMI: 18.5–30 kg/m^2^; Stable weight (previous 3 months) | Pregnancy/breastfeeding |
| **Physical Activity** | Maintenance of habitual physical activity during the study | > 5 h moderate-vigorous exercise/week |
| **Dietary Habits, Supplements** | Omnivore or vegetarian; Dietary fibre intake: 15–25 g/day (evaluated by two food diaries and one 24-h recall) | Protein intake: > 15 % of energy or max. 1.2 g/kg body mass/day (evaluated by two food diaries and one 24-h recall); Diet affecting the study participation/ results; Regular intake of probiotic/prebiotic products (previous 6 months); Intolerance to study products |
| **Medical History, Drugs** |  | Acute/chronic disease, inflammatory/functional gastrointestinal diseases and any other disease/disorder that could affect the study outcome; Medication that may interfere with the study outcome; Eating disorder; Antibiotics and/or laxative/anti-diarrheal medication (previous 3 months); Tobacco/nicotine use; Abuse of alcohol/drugs |

**Online Resource 1** Applied inclusion and exclusion criteria

BMI: Body Mass Index
