# Supplementary material for: Differential effects of pea protein isolate, pea protein concentrate, and whey protein isolate supplementation on gut microbiota and metabolic markers in healthy adults: a 3-arm parallel randomised intervention
Source: Eur J Nutr. 2026 Nov 11;65(8):299. doi: 10.1007/s00394-026-04143-z (PMC13755021; doi:10.1007/s00394-026-04143-z)
Supplement: Supplementary file 4 [file 394_2026_4143_MOESM1_ESM.zip › Online Resource 3_Gut microbiota diversity measures_revision.docx]

**Differential effects of pea protein isolate, pea protein concentrate, and whey protein isolate supplementation on gut microbiota and metabolic markers in healthy adults: a 3-arm parallel randomised intervention**

Annalena Kamm^a,b^, Samira Prado^a,b,c^, Katharina Dannenberg^a,d^, Dirk Repsilber^a,b^, Tatiana M. Marques^a,b^, Robert J. Brummer^a,b^

^a^School of Medical Sciences, Faculty of Medicine and Health, Örebro University, Örebro, Sweden

^b^Food and Health Centre, Örebro University, Örebro, Sweden

^c^School of Science and Technology, Faculty of Business, Science and Engineering, Örebro University, Sweden

^d^Clinical Genomics Örebro, Science for Life Laboratory, Örebro University, Örebro, Sweden

**Corresponding Authors**

Annalena Kamm

annalena.kamm@oru.se

ORCiD: 0009-0002-9389-4172

Samira Prado

samira.prado@oru.se

ORCiD: 0000-0003-4627-6291

**Online Resource 3** Gut microbiota diversity measures including descriptive statistics and results from the main statistical model (LMMs) for each comparison (PPC vs. PPI and PPI vs. WPI)

|  | Visit | PPC |  | PPI |  | WPI |  | PPC vs. PPI | | PPI vs. WPI | |
| --- | --- | --- | --- | --- | --- | --- | --- | --- | --- | --- | --- |
|  |  | *Median (IQR)* | *n* | *Median (IQR)* | *n* | *Median (IQR)* | *n* | *Factor* | *p-value* | *Factor* | *p-value* |
| α-diversity  (Shannon Diversity Index) | 2 | 3.7 (3.5–3.8) | 17 | 4.2 (3.7–4.3) | 18 | 3.7 (3.5–4.1) | 18 | T  TR  BMI  T-BMI  T-TR  TR-BMI  T-TR-BMI | **< 0.0001**  **0.0330**  **0.0002**  0.2722  0.5074  0.8465  0.7113 | T  TR  BMI  T-BMI  T-TR  TR-BMI  T-TR-BMI | **0.0036**  0.1205  **0.0114**  0.4131  0.6595  0.6591  0.7611 |
|  | 4 | 3.8 (3.5–4.0) | 18 | 4.3 (3.7–4.5) | 18 | 3.8 (3.3–4.0) | 17 |  |  |  |  |
|  | 6 | 3.5 (3.3–3.7) | 19 | 4.0 (3.3–4.2) | 19 | 3.7 (3.5–4.0) | 18 |  |  |  |  |
| β-diversity  (Bray-Curtis Index) | 2 vs. 4 | 0.3 (0.2–0.3) | 16 | 0.2 (0.2–0.3) | 17 | 0.2 (0.2–0.3) | 17 | T  TR  BMI  T-BMI  T-TR  TR-BMI  T-TR-BMI | **0.0054**  0.1022  0.3275  0.5533  0.9950  0.2469  0.5668 | T  TR  BMI  T-BMI  T-TR  TR-BMI  T-TR-BMI | **0.0191**  0.5531  0.5793  0.4781  0.3869  0.7691  0.4906 |
|  | 2 vs. 6 | 0.3 (0.3–0.3) | 17 | 0.3 (0.2–0.3) | 18 | 0.3 (0.2–0.3) | 18 |  |  |  |  |

Shannon Diversity Index (α-diversity) was calculated at the sample level from OTU count data,, Bray-Curtis Index (β-diversity) was quantified by calculating pairwise distances for each participant between samples from V2 to V4 as well as V2 to V6; IQR: Interquartile Range, PPC: Pea Protein Concentrate, PPI: Pea Protein Isolate, T: Time, T-BMI: Time-BMI Interaction, T-TR: Time-Treatment Interaction, TR: Treatment, TR-BMI: Treatment-BMI Interaction, T-TR-BMI: Time-Treatment-BMI Interaction, WPI: Whey Protein Isolate; Significances are indicated in bold (descriptive p-values) , α = 0.05
