# Supplementary material for: Differential effects of pea protein isolate, pea protein concentrate, and whey protein isolate supplementation on gut microbiota and metabolic markers in healthy adults: a 3-arm parallel randomised intervention
Source: Eur J Nutr. 2026 Nov 11;65(8):299. doi: 10.1007/s00394-026-04143-z (PMC13755021; doi:10.1007/s00394-026-04143-z)
Supplement: Supplementary file 4 [file 394_2026_4143_MOESM1_ESM.zip › Online Resource 5_Relative abundance data of species with significant interactions (PPC vs. PPI)_revision.docx]

**Differential effects of pea protein isolate, pea protein concentrate, and whey protein isolate supplementation on gut microbiota and metabolic markers in healthy adults: a 3-arm parallel randomised intervention**

Annalena Kamm^a,b^, Samira Prado^a,b,c^, Katharina Dannenberg^a,d^, Dirk Repsilber^a,b^, Tatiana M. Marques^a,b^, Robert J. Brummer^a,b^

^a^School of Medical Sciences, Faculty of Medicine and Health, Örebro University, Örebro, Sweden

^b^Food and Health Centre, Örebro University, Örebro, Sweden

^c^School of Science and Technology, Faculty of Business, Science and Engineering, Örebro University, Sweden

^d^Clinical Genomics Örebro, Science for Life Laboratory, Örebro University, Örebro, Sweden

**Corresponding Authors**

Annalena Kamm

annalena.kamm@oru.se

ORCiD: 0009-0002-9389-4172

Samira Prado

samira.prado@oru.se

ORCiD: 0000-0003-4627-6291

**Online Resource 5** Log-transformed, standardised, and sparsity-reduced relative abundance data of species with significant interactions over the course of the intervention (PPC vs. PPI)

| **Species** | **Relative Abundance**  (Median (IQR)) | | | | | | **p-adj^#^** |
| --- | --- | --- | --- | --- | --- | --- | --- |
|  | PPC | | | PPI | | |  |
|  | *V2* | *V4* | *V6* | *V2* | *V4* | *V6* |  |
| **Time:Treatment Interaction** |  |  |  |  |  |  |  |
| *Anaerobutyricum hallii* | -0.42  (-0.59, 0.10) | 0.31  (-0.12, 0.72) | 0.88  (0.17, 1.69) | -0.18  (-0.42, 0.00) | -0.68  (-0.82, -0.56) | -0.31  (-0.56, 0.39) | 0.0400 |
| *Anaerotignum faecicola* | 0.20  (-0.45, 0.95) | -0.41  (-0.73, 0.31) | -0.61  (-0.73, -0.01) | -0.27  (-0.73, 0.46) | -0.02  (-0.47, 0.82) | -0.43  (-0.73, -0.04) | 0.0391 |
| *Bifidobacterium longum* | -0.35  (-0.48, -0.24) | 0.14  (-0.44, 0.31) | 0.36  (-0.17, 0.72) | -0.32  (-0.37, -0.18) | -0.43  (-0.52, -0.21) | -0.34  (-0.48, -0.18) | 0.0020 |
| *Butyricimonas faecihominis* | -0.25  (-0.25, -0.19) | -0.25  (-0.25, -0.05) | -0.25  (-0.25, -0.25) | -0.25  (-0.25, -0.25) | -0.25  (-0.25, -0.25) | -0.25  (-0.25, -0.25) | 0.0480 |
| *Clostridiaceae unclassified SGB15090* | -0.34  (-0.34, -0.34) | -0.34  (-0.34, -0.34) | -0.34  (-0.34, -0.34) | -0.32  (-0.34, 0.21) | -0.34  (-0.34, -0.02) | -0.34  (-0.34, -0.32) | 0.0400 |
| *Clostridium sp NSJ 42* | -0.22  (-0.22, -0.22) | -0.22  (-0.22, -0.22) | -0.22  (-0.22, -0.22) | -0.22  (-0.22, -0.10) | -0.20  (-0.22, -0.16) | -0.22  (-0.22, -0.19) | 0.0476 |
| *Coprococcus catus* | -0.30  (-0.78, 0.22) | -0.14  (-0.73, 0.57) | 0.12  (-0.61, 0.96) | -0.03  (-0.74, 0.19) | -0.21  (-0.58, 0.14) | -0.15  (-0.36, 0.19) | 0.0474 |
| *Eubacteriales unclassified Clostridiales bacterium KLE1615* | 0.02  (-0.87, 0.59) | -0.29  (-0.93, 0.30) | -0.68  (-1.15, 0.23) | 0.31  (-0.02, 0.50) | 0.14  (-0.41, 0.25) | 0.50  (-0.08, 1.18) | 0.0400 |
| *Faecalicatena fissicatena* | -0.04  (-0.63, 0.63) | -0.42  (-0.59, -0.01) | -0.72  (-0.80, 0.40) | 0.26  (-0.72, 0.82) | -0.01  (-0.70, 0.96) | 0.30  (-0.70, 1.24) | 0.0480 |
| *Flavonifractor plautii* | -0.14  (-0.26, -0.01) | -0.24  (-0.28, -0.15) | -0.28  (-0.28, -0.28) | -0.28  (-0.28, -0.28) | -0.28  (-0.28, -0.28) | -0.28  (-0.28, -0.28) | 0.0474 |
| *GGB3277 SGB4327* | -0.45  (-0.45, -0.45) | -0.45  (-0.45, -0.45) | -0.45  (-0.45, -0.45) | -0.45  (-0.45, 0.58) | -0.45  (-0.45, 1.82) | -0.45  (-0.45, 1.04) | 0.0476 |
| *GGB4684 SGB6478* | -0.25  (-0.25, -0.22) | -0.25  (-0.25, -0.25) | -0.25  (-0.25, -0.24) | -0.25  (-0.25, -0.25) | -0.25  (-0.25, 0.07) | -0.25  (-0.25, -0.17) | 0.0474 |
| *GGB4750 SGB6579* | -0.24  (-0.24, -0.24) | -0.24  (-0.24, -0.24) | -0.24  (-0.24 -0.24) | -0.24  (-0.24, -0.16) | -0.24  (-0.24, 0.29) | -0.24  (-0.24, -0.24) | 0.0474 |
| *GGB9641 SGB15117* | -0.31  (-0.31, -0.30) | -0.31  (-0.31, -0.31) | -0.31  (-0.31, -0.24) | -0.31  (-0.31, -0.31) | -0.31  (-0.31, -0.16) | -0.31  (-0.31, -0.31) | 0.0364 |
| *Lactobacillus crispatus* | -0.19  (-0.19, -0.19) | -0.19  (-0.19, -0.19) | -0.19  (-0.19, -0.19) | -0.19  (-0.19, -0.19) | -0.19  (-0.19, 0.63) | -0.19  (-0.19, 0.56) | 0.0155 |
| *Lawsonibacter sp NSJ 51* | -0.18  (-0.18, -0.18) | -0.18  (-0.18, -0.12) | -0.18  (-0.18, -0.18) | -0.18  (-0.18, -0.18) | -0.18  (-0.18, -0.18) | -0.18  (-0.18, -0.18) | 0.0400 |
| *Limosilactobacillus frumenti* | -0.26  (-0.26, -0.26) | -0.26  (-0.26, -0.26) | -0.26  (-0.26, -0.26) | -0.26  (-0.26, -0.26) | 0.28  (-0.12, 0.90) | 0.05  (-0.26, 1.34) | <0.0001 |
| *Mediterraneibacter torques* | -0.13  (-0.33, 0.30) | -0.36  (-0.58, -0.05) | -0.34  (-0.50, 0.02) | -0.29  (-0.44, -0.16) | -0.33  (-0.45, -0.26) | -0.25  (-0.43, 0.36) | 0.0480 |
| *Negativibacillus massiliensis* | -0.21  (-0.26, 0.37) | -0.26  (-0.26, -0.02) | -0.26  (-0.26, -0.25) | -0.26  (-0.26, -0.26) | -0.26  (-0.26, -0.26) | -0.26  (-0.26, -0.26) | 0.0474 |
| *Odoribacter splanchnicus* | -0.05  (-0.38, 0.46) | 0.01  (-0.18, 0.84) | -0.56  (-0.67, -0.49) | -0.31  (-0.59, -0.16) | 0.62  (-0.32, 2.38) | -0.59  (-0.67, -0.16) | 0.0400 |
| *Oscillibacter sp ER4* | 0.55  (-0.35, 1.04) | 0.09  (-0.86, 0.91) | -0.73  (-0.92, -0.23) | -0.28  (-0.91, 0.43) | -0.39  (-0.74, 0.77) | -0.53  (-0.92, 0.50) | 0.0474 |
| *Rheinheimera SGB14999* | 0.12  (-0.45, 0.54) | -0.47  (-0.49, -0.11) | -0.49  (-0.49, -0.48) | -0.49  (-0.49, -0.49) | -0.49  (-0.49, -0.43) | -0.49  (-0.49, -0.49) | 0.0400 |
| **Time:Treatment:BMI Interaction** |  |  |  |  |  |  |  |
| *Alistipes ihumii* | -0.22  (-0.54, 1.42) | -0.54  (-0.54, -0.54) | -0.41  (-0.54, 0.49) | -0.54  (-0.54, -0.09) | -0.46  (-0.54, 0.49) | -0.54  (-0.54, -0.22) | 0.0480 |
| *Bacteroides fragilis* | -0.16  (-0.16, -0.09) | -0.16  (-0.16, -0.16) | -0.16  (-0.16, -0.16) | -0.16  (-0.16, -0.13) | -0.14  (-0.16, 0.24) | -0.16  (-0.16, -0.15) | 0.0400 |
| *Clostridiaceae unclassified Clostridiaceae bacterium Marseille Q4145* | -0.30  (-0.38, 0.29) | -0.37  (-0.38, -0.04) | -0.38  (-0.38, -0.24) | -0.35  (-0.38, 0.62) | -0.01  (-0.38, 0.21) | -0.25  (-0.38, 0.27) | 0.0400 |
| *Clostridiales Family XIII bacterium BX16* | -0.41  (-0.59, 0.57) | -0.32  (-0.60, 0.28) | -0.31  (-0.75, 0.18) | -0.30  (-0.74, 0.60) | -0.06  (-0.52, 0.25) | -0.38  (-0.74, 0.31) | 0.0474 |
| *Desulfovibrio piger* | -0.33  (-0.33, -0.33) | -0.33  (-0.33, -0.33) | -0.33  (-0.33, -0.33) | -0.33  (-0.33, -0.22) | -0.33  (-0.33, -0.33) | -0.33  (-0.33, 0.37) | 0.0474 |
| *Eubacterium ventriosum* | 0.03  (-0.42, 0.54) | -0.71  (-0.78, -0.29) | -0.50  (-0.72, 0.26) | 0.03  (-0.77, 0.59) | 0.59  (-0.24, 1.01) | -0.48  (-0.78, 0.12) | 0.0400 |
| *GGB10692 SGB17347* | -0.27  (-0.27, -0.24) | -0.27  (-0.27, -0.27) | -0.27  (-0.27, -0.24) | -0.27  (-0.27, -0.08) | -0.27  (-0.27, -0.27) | -0.27  (-0.27, 0.29) | 0.0474 |
| *GGB2983 SGB3965* | -0.30  (-0.30, -0.30) | -0.30  (-0.30, -0.30) | -0.30  (-0.30, -0.30) | -0.30  (-0.30, -0.30) | -0.30  (-0.30, -0.30) | -0.30  (-0.30, -0.30) | 0.0497 |
| *GGB2998 SGB3988* | -0.50  (-0.50, 0.41) | -0.50  (-0.50, -0.20) | -0.50  (-0.50, -0.50) | -0.50  (-0.50, 0.44) | -0.47  (-0.50, 1.05) | -0.32  (-0.50, 0.39) | 0.0400 |
| *GGB33469 SGB15236* | -0.49  (-0.50, 0.17) | -0.46  (-0.50, 0.26) | -0.50  (-0.50, -0.37) | -0.07  (-0.47, 0.73) | -0.50  (-0.50, -0.18) | -0.21  (-0.50, 2.00) | 0.0474 |
| *GGB74463 SGB54347* | -0.13  (-0.13, -0.13) | -0.13  (-0.13, -0.13) | -0.13  (-0.13, -0.13) | -0.13  (-0.13, -0.13) | -0.13  (-0.13, -0.13) | -0.13  (-0.13, -0.13) | 0.0480 |
| *GGB9063 SGB13982* | -0.23  (-0.23, -0.20) | -0.23  (-0.23, -0.23) | -0.23  (-0.23, -0.23) | -0.23  (-0.23, -0.23) | -0.23  (-0.23, -0.23) | -0.23  (-0.23, -0.23) | 0.0480 |
| *GGB9708 SGB15234* | -0.42  (-0.42, -0.27) | -0.42  (-0.42, 0.18) | -0.42  (-0.42, -0.40) | -0.30  (-0.42, 0.25) | -0.42  (-0.42, -0.18) | -0.18  (-0.42, 0.12) | 0.0400 |
| *GGB9775 SGB15395* | -0.28  (-0.28, -0.28) | -0.28  (-0.28, -0.28) | -0.28  (-0.28, -0.28) | -0.28  (-0.28, -0.13) | -0.28  (-0.28, -0.28) | -0.28  (-0.28, -0.28) | 0.0474 |
| *GGB9786 SGB15409* | -0.33  (-0.33, -0.19) | -0.33  (-0.33, 0.69) | -0.33  (-0.33, -0.33) | -0.33  (-0.33, -0.15) | -0.33  (-0.33, -0.33) | -0.33  (-0.33, -0.33) | 0.0400 |
| *Lacrimispora amygdalina* | 0.18  (-0.50, 0.58) | -0.46  (-0.53, -0.07) | -0.50  (-0.53, -0.43) | -0.43  (-0.53, -0.03) | -0.15  (-0.46, 0.38) | -0.24  (-0.49, 0.17) | 0.0474 |
| *Lacrimispora celerecrescens* | -0.49  (-0.83, -0.16) | 0.37  (-0.66, 0.56) | -0.31  (-0.71, 0.04) | -0.09  (-0.40, 0.38) | -0.25  (-0.88, 0.22) | -0.09  (-0.71, 0.36) | 0.0400 |
| *Lactobacillus delbrueckii* | -0.24  (-0.24, -0.24) | -0.24  (-0.24, 0.12) | -0.24  (-0.24, -0.24) | -0.24  (-0.24, -0.24) | -0.24  (-0.24, -0.24) | -0.24  (-0.24, -0.24) | 0.0497 |
| *Massiliimalia timonensis* | -0.35  (-0.35, 0.28) | -0.35  (-0.35, -0.35) | -0.35  (-0.35, -0.33) | -0.35  (-0.35, -0.35) | -0.35  (-0.35, -0.16) | -0.35  (-0.35 -0.35) | 0.0400 |
| *Massilimicrobiota timonensis* | -0.24  (-0.24, 0.03) | -0.24  (-0.24, -0.24) | -0.24  (-0.24, -0.16) | -0.24  (-0.24, -0.24) | -0.24  (-0.24, -0.24) | -0.24  (-0.24, -0.24) | 0.0400 |
| *Mogibacterium sp BX12* | -0.41  (-0.41, 0.39) | -0.41  (-0.41, -0.30) | -0.41  (-0.41, -0.10) | -0.41  (-0.41, -0.33) | -0.41  (-0.41, -0.25) | -0.41  (-0.41, -0.37) | 0.0480 |

Sparsity reduction was performed by removing all taxa whose sum across samples was within the bottom 30% of sums, as well as all taxa that appeared in fewer than two samples. Data were log-transformed using log1p(x) and standardised to a mean of zero and a standard deviation of one for each taxon across samples. IQR: Interquartile Range, PPC: Pea Protein Concentrate, PPI: Pea Protein Isolate, **^#^**p-values were adjusted using the Benjamini-Hochberg correction, α = 0.05
