# Supplementary material for: Differential effects of pea protein isolate, pea protein concentrate, and whey protein isolate supplementation on gut microbiota and metabolic markers in healthy adults: a 3-arm parallel randomised intervention
Source: Eur J Nutr. 2026 Nov 11;65(8):299. doi: 10.1007/s00394-026-04143-z (PMC13755021; doi:10.1007/s00394-026-04143-z)
Supplement: Supplementary file 4 [file 394_2026_4143_MOESM1_ESM.zip › Online Resource 6_Relative abundance data of species with significant interactions (PPI vs. WPI)_revision.docx]

**Differential effects of pea protein isolate, pea protein concentrate, and whey protein isolate supplementation on gut microbiota and metabolic markers in healthy adults: a 3-arm parallel randomised intervention**

Annalena Kamm^a,b^, Samira Prado^a,b,c^, Katharina Dannenberg^a,d^, Dirk Repsilber^a,b^, Tatiana M. Marques^a,b^, Robert J. Brummer^a,b^

^a^School of Medical Sciences, Faculty of Medicine and Health, Örebro University, Örebro, Sweden

^b^Food and Health Centre, Örebro University, Örebro, Sweden

^c^School of Science and Technology, Faculty of Business, Science and Engineering, Örebro University, Sweden

^d^Clinical Genomics Örebro, Science for Life Laboratory, Örebro University, Örebro, Sweden

**Corresponding Authors**

Annalena Kamm

annalena.kamm@oru.se

ORCiD: 0009-0002-9389-4172

Samira Prado

samira.prado@oru.se

ORCiD: 0000-0003-4627-6291

**Online Resource 6** Log-transformed, standardised, and sparsity-reduced relative abundance data of species with significant interactions over the course of the intervention (PPI vs. WPI)

| **Species** | **Relative Abundance**  (Median (IQR)) | | | | | | **p-adj^#^** |
| --- | --- | --- | --- | --- | --- | --- | --- |
|  | PPI | | | WPI | | |  |
|  | *V2* | *V4* | *V6* | *V2* | *V4* | *V6* |  |
| **Time:Treatment Interaction** |  |  |  |  |  |  |  |
| *Acidaminococcus intestini* | -0.22  (-0.22, -0.22) | -0.22  (-0.22, -0.22) | -0.22  (-0.22, -0.22) | -0.22  (-0.22, 0.46) | -0.22  (-0.22, -0.04) | -0.22  (-0.22, -0.14) | 0.0430 |
| *Alistipes sp AF17 16* | -0.37  (-0.37, -0.37) | -0.37  (-0.37, -0.09) | -0.37  (-0.37, -0.37) | -0.37  (-0.37, -0.02) | -0.37  (-0.37, -0.36) | -0.37  (-0.37, -0.07) | 0.0430 |
| *Anaerobutyricum hallii* | -0.18  (-0.42, 0.00) | -0.68  (-0.82, -0.56) | -0.31  (-0.56, 0.39) | -0.61  (-0.95, -0.09) | -0.14  (-0.76, 0.26) | -0.34  (-0.94, 1.07) | 0.0428 |
| *Anaerotignum faecicola* | -0.27  (-0.73, 0.46) | -0.02  (-0.47, 0.82) | -0.43  (-0.73, -0.04) | -0.65  (-0.73, 0.08) | -0.70  (-0.73, -0.08) | -0.66  (-0.73, 0.02) | 0.0313 |
| *Candidatus Avimonas narfia* | -0.44  (-0.44, -0.37) | -0.44  (-0.44, 0.01) | -0.44  (-0.44, -0.44) | -0.44  (-0.44, -0.06) | -0.44  (-0.44, -0.35) | -0.38  (-0.44, -0.02) | 0.0462 |
| *Clostridiaceae unclassified SGB15090* | -0.32  (-0.34, 0.21) | -0.34  (-0.34, -0.02) | -0.34  (-0.34, -0.32) | -0.34  (-0.34, -0.30) | -0.34  (-0.34, -0.28) | -0.34  (-0.34, -0.29) | 0.0401 |
| *Clostridium SGB6179* | -0.34  (-0.35, 0.02) | -0.35  (-0.35, 0.66) | -0.35  (-0.35, -0.31) | -0.35  (-0.35, -0.32) | -0.35  (-0.35, -0.35) | -0.35  (-0.35, -0.01) | 0.0401 |
| *Clostridium sp 1001270H 150608 G6* | -0.33  (-0.33, 0.06) | -0.33  (-0.33 -0.29) | -0.33  (-0.33, -0.31) | -0.33  (-0.33, -0.26) | -0.33  (-0.33, -0.31) | -0.33  (-0.33, -0.28) | 0.0430 |
| *Eubacteriales unclassified Clostridiales bacterium KLE1615* | 0.31  (-0.02, 0.50) | 0.14  (-0.41, 0.25) | 0.50  (-0.08, 1.18) | -0.37  (-0.91, 0.61) | -0.52  (-0.89, 0.45) | -0.67  (-1.16, -0.17) | 0.0274 |
| *Eubacteriales unclassified Clostridiales bacterium UBA1390* | -0.32  (-0.32, -0.32) | -0.32  (-0.32, -0.32) | -0.32  (-0.32, -0.32) | -0.32  (-0.32, -0.32) | -0.32  (-0.32, -0.32) | -0.32  (-0.32, -0.32) | 0.0430 |
| *Eubacteriales unclassified SGB15145* | -0.26  (-0.26, 0.07) | 0.00  (-0.26, 1.21) | -0.26  (-0.26, -0.17) | -0.26  (-0.26, -0.26) | -0.26  (-0.26, -0.26) | -0.26  (-0.26, -0.26) | 0.0406 |
| *Eubacterium ventriosum* | 0.03  (-0.77, 0.59) | -0.48  (-0.78, 0.12) | -0.71  (-0.78, -0.29) | -0.55  (-0.78, 0.47) | -0.74  (-0.78, 0.30) | -0.75  (-0.78, -0.26) | 0.0430 |
| *GGB2980 SGB3962* | -0.36  (-0.36, -0.36) | -0.36  (-0.36, -0.36) | -0.36  (-0.36, -0.36) | -0.36  (-0.36, -0.29) | -0.36  (-0.36, -0.29) | -0.36  (-0.36, 0.41) | 0.0430 |
| *GGB2998 SGB3988* | -0.50  (-0.50, 0.44) | -0.32  (-0.50, 0.39) | -0.50  (-0.50, -0.20) | -0.50  (-0.50, -0.37) | -0.50  (-0.50, -0.50) | -0.50  (-0.50, -0.38) | 0.0490 |
| *GGB3175 SGB4191* | -0.28  (-0.39, 0.02) | 0.12  (-0.39, 0.80) | -0.30  (-0.39, 0.17) | -0.39  (-0.39, -0.16) | -0.39  (-0.39, -0.30) | -0.39  (-0.39, -0.02) | 0.0430 |
| *GGB3304 SGB4367* | -0.44  (-0.45, -0.04) | -0.44  (-0.45, 0.63) | -0.45  (-0.45, 0.16) | -0.45  (-0.45, 0.39) | -0.45  (-0.45, -0.45) | -0.45  (-0.45, -0.45) | 0.0401 |
| *GGB33928 SGB15225* | -0.21  (-0.51, 0.45) | -0.04  (-0.51 0.83) | -0.51  (-0.51, -0.15) | -0.51  (-0.51, -0.37) | -0.51  (-0.51, -0.36) | -0.51  (-0.51, -0.49) | 0.0430 |
| *GGB4768 SGB6601* | -0.25  (-0.25, 0.03) | -0.13  (-0.25, 0.48) | -0.25  (-0.25, -0.23) | -0.25  (-0.25, -0.25) | -0.25  (-0.25, -0.25) | -0.25  (-0.25, -0.25) | 0.0401 |
| *GGB80140 SGB15224* | -0.41  (-0.48, 0.33) | -0.30  (-0.48, 1.26) | -0.48  (-0.48, 0.30) | -0.48  (-0.48, -0.48) | -0.48  (-0.48, -0.48) | -0.48  (-0.48, -0.46) | 0.0401 |
| *GGB9261 SGB14209* | -0.25  (-0.25, -0.16) | -0.25  (-0.25, 0.68) | -0.25  (-0.25, -0.25) | -0.25  (-0.25, -0.25) | -0.25  (-0.25, -0.25) | -0.25  (-0.25, -0.25) | 0.0441 |
| *GGB9345 SGB14311* | -0.15  (-0.45, 0.77) | 0.28  (-0.45, 1.49) | -0.27  (-0.45, 0.41) | -0.45  (-0.45, -0.44) | -0.45  (-0.45, -0.38) | -0.45  (-0.45, -0.29) | 0.0490 |
| *GGB9522 SGB14921* | 0.00  (-0.58, 1.11) | -0.31  (-0.58, 1.35) | -0.53  (-0.58, 0.77) | -0.58  (-0.58, -0.47) | -0.58  (-0.58, -0.58) | -0.58  (-0.58, 0.57) | 0.0334 |
| *GGB9602 SGB15031* | -0.28  (-0.46, 0.41) | -0.32  (-0.46, 0.18) | -0.45  (-0.46, -0.11) | -0.46  (-0.46, 0.08) | -0.46  (-0.46, 0.30) | -0.46  (-0.46, 0.29) | 0.0448 |
| *GGB9634 SGB15093* | -0.31  (-0.31, -0.02) | -0.29  (-0.31, 0.15) | -0.31  (-0.31, -0.28) | -0.31  (-0.31, -0.31) | -0.31  (-0.31, -0.31) | -0.31  (-0.31, -0.31) | 0.0401 |
| *GGB9707 SGB15229* | -0.08  (-0.39, 0.72) | -0.01  (-0.44, 0.99) | -0.27  (-0.60, 0.13) | -0.60  (-0.60, 0.18) | -0.60  (-0.60, -0.27) | -0.60  (-0.60, -0.43) | 0.0430 |
| *GGB9770 SGB15390* | -0.31  (-0.31, -0.26) | -0.31  (-0.31, -0.31) | -0.31  (-0.31 -0.31) | -0.31  (-0.31, -0.27) | -0.31  (-0.31, 0.11) | -0.31  (-0.31, 0.06) | 0.0401 |
| *Lacrimispora saccharolytica* | -0.15  (-0.15, -0.15) | -0.15  (-0.15, -0.15) | -0.15  (-0.15, -0.15) | -0.15  (-0.15, -0.15) | -0.15  (-0.15, 0.01) | -0.15  (-0.15, -0.15) | 0.0441 |
| *Lactobacillus acidophilus* | -0.15  (-0.15, -0.15) | -0.15  (-0.15, -0.15) | -0.19  (-0.19, -0.19) | -0.15  (-0.15, -0.15) | -0.15  (-0.15, -0.15) | -0.15  (-0.15, -0.15) | 0.0401 |
| *Lactobacillus crispatus* | -0.19  (-0.19, -0.19) | -0.19  (-0.19, 0.63) | -0.19  (-0.19, 0.56) | -0.19  (-0.19, -0.19) | -0.19  (-0.19, -0.19) | -0.19  (-0.19, -0.19) | 0.0313 |
| *Limosilactobacillus frumenti* | -0.26  (-0.26, -0.26) | 0.28  (-0.12, 0.90) | 0.05  (-0.26, 1.34) | -0.26  (-0.26, -0.26) | -0.26  (-0.26, -0.26) | -0.26  (-0.26, -0.26) | <0.0001 |
| *Mediterraneibacter butyricigenes* | -0.33  (-0.52, 0.01) | -0.16  (-0.48, 0.37) | -0.56  (-0.74 -0.21) | -0.28  (-0.84, 1.09) | -0.30  (-0.84, 0.85) | 0.02  (-0.84, 0.14) | 0.0462 |
| *Mediterraneibacter lactaris* | -0.26  (-0.49, 0.24) | -0.02  (-0.49, 0.49) | -0.23  (-0.49, 0.22) | -0.49  (-0.49, -0.45) | -0.49  (-0.49, -0.36) | -0.49  (-0.49, 0.40) | 0.0462 |
| *Ruthenibacterium lactatiformans* | -0.27  (-0.29, -0.20) | -0.26  (-0.30, -0.17) | -0.29  (-0.30, -0.26) | -0.12  (-0.27, -0.09) | -0.23  (-0.30, -0.03) | -0.11  (-0.29, 0.17) | 0.0448 |
| *Slackia isoflavoniconvertens* | -0.47  (-0.47, 0.09) | -0.47  (-0.47, 0.16) | -0.40  (-0.47, 1.38) | -0.47  (-0.47, -0.47) | -0.47  (-0.47, -0.47) | -0.47  (-0.47, -0.47) | 0.0490 |
| *Vescimonas coprocola* | -0.35  (-0.40, -0.23) | -0.29  (-0.36, -0.15) | -0.39  (-0.42, -0.27) | -0.33  (-0.42, -0.21) | -0.15  (-0.42, 0.16) | -0.23  (-0.39, 0.02) | 0.0401 |
| *Vescimonas SGB15087* | -0.28  (-0.28, -0.13) | -0.26  (-0.28, 0.06) | -0.28  (-0.28, -0.07) | -0.28  (-0.28, -0.28) | -0.28  (-0.28, -0.28) | -0.28  (-0.28, -0.28) | 0.0462 |
| **Time:Treatment:BMI Interaction** |  |  |  |  |  |  |  |
| *Bacteroides xylanisolvens* | -0.46  (-0.46, -0.34) | -0.43  (-0.46, -0.07) | -0.46  (-0.46, -0.30) | -0.26  (-0.46, 0.99) | -0.12  (-0.46, 0.24) | -0.33  (-0.46, -0.02) | 0.0462 |
| *Bifidobacterium longum* | -0.32  (-0.37, -0.18) | -0.43  (-0.52, -0.21) | -0.34  (-0.48, -0.18) | -0.29  (-0.37, 0.10) | -0.25  (-0.38, 0.14) | -0.27  (-0.36, 0.04) | 0.0462 |
| *Coprococcus catus* | -0.03  (-0.74, 0.19) | -0.21  (-0.58, 0.14) | -0.15  (-0.36, 0.19) | 0.04  (-0.97, 0.50) | -0.08  (-0.72, 0.42) | 0.46  (-0.04, 1.62) | 0.0401 |
| *GGB2982 SGB3964* | -0.01  (-0.71, 0.83) | -0.24  (-0.71, 1.34) | -0.71  (-0.71, -0.36) | -0.38  (-0.71, 0.30) | -0.38  (-0.71, 0.29) | 0.02  (-0.71, 0.96) | 0.0490 |
| *GGB3034 SGB4030* | -0.33  (-0.33, -0.33) | -0.33  (-0.33, -0.33) | -0.33  (-0.33, -0.33) | -0.33  (-0.33, -0.33) | -0.33  (-0.33, -0.33) | -0.33  (-0.33, -0.33) | 0.0430 |
| *GGB4703 SGB6510* | -0.20  (-0.20, -0.20) | -0.20  (-0.20, 0.42) | -0.20  (-0.20, -0.20) | -0.20  (-0.20, -0.20) | -0.20  (-0.20, -0.20) | -0.20  (-0.20, -0.20) | 0.0460 |
| *GGB4750 SGB6579* | -0.24  (-0.24, -0.16) | -0.24  (-0.24, 0.29) | -0.24  (-0.24, -0.24) | -0.24  (-0.24, -0.24) | -0.24  (-0.24, -0.24) | -0.24  (-0.24, -0.24) | 0.0430 |
| *GGB9608 SGB15041* | -0.60  (-0.60, 0.40) | -0.58  (-0.60, 0.61) | -0.56  (-0.60, 0.78) | -0.60  (-0.60, -0.59) | -0.60  (-0.60, -0.26) | -0.60  (-0.60, -0.53) | 0.0430 |
| *GGB9642 SGB15119* | -0.42  (-0.42, -0.42) | -0.42  (-0.42, -0.42) | -0.42  (-0.42, -0.42) | -0.42  (-0.42, -0.42) | -0.42  (-0.42, -0.42) | -0.42  (-0.42, -0.42) | 0.0430 |
| *Lachnospira pectinoschiza* | -0.24  (-0.50, 0.25) | -0.11  (-0.50, 0.14) | -0.42  (-0.50, -0.08) | -0.23  (-0.50, 0.46) | -0.18  (-0.50, 1.17) | -0.49  (-0.50, -0.39) | 0.0401 |
| *Lawsonibacter sp NSJ 51* | -0.18  (-0.18, -0.18) | -0.18  (-0.18, -0.18) | -0.18  (-0.18, -0.18) | -0.18  (-0.18, -0.18) | -0.18  (-0.18, -0.18) | -0.18  (-0.18, -0.18) | 0.0462 |
| *Mediterraneibacter massiliensis* | -0.27  (-0.27, 0.18) | -0.27  (-0.27, -0.12) | -0.27  (-0.27, -0.27) | -0.27  (-0.27, -0.27) | -0.27  (-0.27, -0.27) | -0.27  (-0.27, -0.27) | 0.0430 |

Sparsity reduction was performed by removing all taxa whose sum across samples was within the bottom 30% of sums, as well as all taxa that appeared in fewer than two samples. Data were log-transformed using log1p(x) and standardised to a mean of zero and a standard deviation of one for each taxon across samples. IQR: Interquartile Range, PPI: Pea Protein Isolate, WPI: Whey Protein Isolate, **^#^**p-values were adjusted using the Benjamini-Hochberg correction
