# Supplementary material for: Differential effects of pea protein isolate, pea protein concentrate, and whey protein isolate supplementation on gut microbiota and metabolic markers in healthy adults: a 3-arm parallel randomised intervention
Source: Eur J Nutr. 2026 Nov 11;65(8):299. doi: 10.1007/s00394-026-04143-z (PMC13755021; doi:10.1007/s00394-026-04143-z)
Supplement: Supplementary file 4 [file 394_2026_4143_MOESM1_ESM.zip › Online Resource 7_Anthropometric measures and dietary intake_revision.docx]

**Differential effects of pea protein isolate, pea protein concentrate, and whey protein isolate supplementation on gut microbiota and metabolic markers in healthy adults: a 3-arm parallel randomised intervention**

Annalena Kamm^a,b^, Samira Prado^a,b,c^, Katharina Dannenberg^a,d^, Dirk Repsilber^a,b^, Tatiana M. Marques^a,b^, Robert J. Brummer^a,b^

^a^School of Medical Sciences, Faculty of Medicine and Health, Örebro University, Örebro, Sweden

^b^Food and Health Centre, Örebro University, Örebro, Sweden

^c^School of Science and Technology, Faculty of Business, Science and Engineering, Örebro University, Sweden

^d^Clinical Genomics Örebro, Science for Life Laboratory, Örebro University, Örebro, Sweden

**Corresponding Authors**

Annalena Kamm

annalena.kamm@oru.se

ORCiD: 0009-0002-9389-4172

Samira Prado

samira.prado@oru.se

ORCiD: 0000-0003-4627-6291

**Online Resource 7** Anthropometric measures and dietary intake during the intervention period

|  | **Visit** | **PPC (n=19)** | **PPI (n=19)** | **WPI (n=18)** | **p-value** | | | | | |
| --- | --- | --- | --- | --- | --- | --- | --- | --- | --- | --- |
|  |  | *Median (IQR)* | | | *PPC vs. PPI* | | | *PPI vs. WPI* | | |
|  |  |  |  |  | *T* | *TR* | *T-TR* | *T* | *TR* | *T-TR* |
| **Anthropometric Measures** |  |  |  |  |  |  |  |  |  |  |
| Body Mass (kg) | 2 | 70.2  (64.9–77.4) | 62.9  (59.8–69.4) | 67.0  (59.5–69.8) | **0.0205** | 0.0709 | 0.0695 | **0.0029** | 0.4833 | 0.4835 |
|  | 4 | 70.0  (64.8–76.4) | 64.6  (59.9–70.4) | 67.2  (60.0–71.3) |  |  |  |  |  |  |
|  | 6 | 69.5  (65.7–77.0) | 65.0  (60.4–69.9) | 66.6  (59.8–71.8) |  |  |  |  |  |  |
| Body Mass Index (kg/m^2^) | 2 | 23.5  (22.5–24.6) | 22.8  (21.1–24.5) | 23.2  (20.8–24.2) | **0.0334** | 0.2124 | 0.2552 | **0.0194** | 0.8711 | 0.5006 |
|  | 4 | 23.7  (22.6–24.9) | 22.6  (20.6–24.3) | 23.1  (20.8–24.2) |  |  |  |  |  |  |
|  | 6 | 23.9  (22.8–24.8) | 23.4  (21.1–24.7) | 23.2  (20.7–24.3) |  |  |  |  |  |  |
| **Dietary Intake** |  |  |  |  |  |  |  |  |  |  |
| Energy (kcal/day) | 2 | 1750.3  (1532.4–2200.0) | 1652.3  (1516.9–2030.7) | 1706.2  (1514.1–1887.4) | **0.0263** | 0.8544 | 0.7274 | **0.0235** | 0.3103 | 0.9892 |
|  | 4 | 1906.1  (1608.5–2510.2) | 1970.2  (1617.3–2258.3) | 1834.9  (1641.6–2148.4) |  |  |  |  |  |  |
|  | 6 | 1841.0  (1577.2–2218.7) | 1843.1  (1624.7–2152.9) | 1642.8  (1410.3–2191.2) |  |  |  |  |  |  |
| Fibre (g/day) | 2 | 16.7  (13.8–21.2) | 18.6  (15.7–23.0) | 18.6  (13.3–23.1) | **0.0148** | 0.4359 | 0.1055 | 0.5045 | 0.4525 | 0.7203 |
|  | 4 | 21.9  (19.4–28.9) | 18.5  (14.5–26.4) | 19.1  (15.7–25.9) |  |  |  |  |  |  |
|  | 6 | 23.5  (18.0–28.7) | 18.4  (14.4–23.4) | 16.0  (14.3–23.6) |  |  |  |  |  |  |
| Carbohydrates (E%) | 2 | 45.5  (42.2–51.1) | 48.0  (41.9–50.3) | 46.2  (45.1–50.5) | **0.0004** | 0.5034 | 0.4499 | **0.0003** | 0.3745 | 0.9828 |
|  | 4 | 40.2  (38.2–46.3) | 41.0  (38.5–45.3) | 42.9  (39.4–44.2) |  |  |  |  |  |  |
|  | 6 | 44.1  (41.0–48.2) | 43.2  (37.3–46.2) | 43.9  (38.6–47.2) |  |  |  |  |  |  |
| Fat (E%) | 2 | 38.3  (33.4–39.8) | 37.2  (32.5–40.9) | 36.5  (33.7–40.1) | **0.0425** | 0.4020 | 0.2104 | 0.2606 | 0.3454 | 0.5585 |
|  | 4 | 36.0  (30.7–39.5 | 35.5  (32.3–38.2) | 34.5  (31.9–36.5) |  |  |  |  |  |  |
|  | 6 | 32.7  (28.8–35.9) | 33.2  (31.2–39.2) | 33.5  (30.4–37.6) |  |  |  |  |  |  |
| Protein (E%) | 2 | 15.0  (13.0–17.4) | 15.9  (14.1–17.8) | 14.6  (13.2–16.1) | **< 0.0001** | 0.6979 | 0.4101 | **< 0.0001** | 0.4307 | 0.5826 |
|  | 4 | 22.4  (18.3–24.5) | 21.0  (19.9–23.1) | 20.9  (19.3–23.7) |  |  |  |  |  |  |
|  | 6 | 22.5  (19.4–24.9) | 23.7  (18.6–24.9) | 22.2  (19.8–24.3) |  |  |  |  |  |  |
| Protein (g/kg body mass/day) | 2 | 0.9  (0.7–1.2) | 1.0  (0.9–1.2) | 0.9  (0.7–1.2) | **< 0.0001** | 0.1086 | 0.4091 | **< 0.0001** | **0.0360** | 0.5268 |
|  | 4 | 1.3  (1.2–1.7) | 1.5  (1.4–1.8) | 1.4  (1.4–1.5) |  |  |  |  |  |  |
|  | 6 | 1.4  (1.1–1.7) | 1.5  (1.4–1.7) | 1.4  (1.1–1.7) |  |  |  |  |  |  |

IQR: Interquartile Range, PPC: Pea Protein Concentrate, PPI: Pea Protein Isolate, T: Time, TR: Treatment, T-TR: Time-Treatment Interaction, WPI: Whey Protein Isolate; Significances are indicated in bold (descriptive p-values originating from a linear mixed-effects model with the factors time, treatment, and their interactions); α = 0.05
