# Supplementary material for: Differential effects of pea protein isolate, pea protein concentrate, and whey protein isolate supplementation on gut microbiota and metabolic markers in healthy adults: a 3-arm parallel randomised intervention
Source: Eur J Nutr. 2026 Nov 11;65(8):299. doi: 10.1007/s00394-026-04143-z (PMC13755021; doi:10.1007/s00394-026-04143-z)
Supplement: Supplementary file 4 [file 394_2026_4143_MOESM1_ESM.zip › Online Resource 8_Physical activity_revision.docx]

**Differential effects of pea protein isolate, pea protein concentrate, and whey protein isolate supplementation on gut microbiota and metabolic markers in healthy adults: a 3-arm parallel randomised intervention**

Annalena Kamm^a,b^, Samira Prado^a,b,c^, Katharina Dannenberg^a,d^, Dirk Repsilber^a,b^, Tatiana M. Marques^a,b^, Robert J. Brummer^a,b^

^a^School of Medical Sciences, Faculty of Medicine and Health, Örebro University, Örebro, Sweden

^b^Food and Health Centre, Örebro University, Örebro, Sweden

^c^School of Science and Technology, Faculty of Business, Science and Engineering, Örebro University, Sweden

^d^Clinical Genomics Örebro, Science for Life Laboratory, Örebro University, Örebro, Sweden

**Corresponding Authors**

Annalena Kamm

annalena.kamm@oru.se

ORCiD: 0009-0002-9389-4172

Samira Prado

samira.prado@oru.se

ORCiD: 0000-0003-4627-6291

**Online Resource 8** Physical activity during the whole study period evaluated using the International Physical Activity Questionnaire

|  | Week | PPC (n=19) | PPI (n=19) | WPI (n=18) | p-value | | | | | |
| --- | --- | --- | --- | --- | --- | --- | --- | --- | --- | --- |
|  |  | *Median (IQR)* | | | *PPC vs. PPI* | | | *PPI vs. WPI* | | |
|  |  |  |  |  | *T* | *TR* | *T-TR* | *T* | *TR* | *T-TR* |
| MET-minutes (week) | 1 | 1440 (783–2465) | 1773 (590–2060) | 1491 (1072–2352) | 0.8193 | 0.9417 | 0.7389 | 0.3118 | 0.3857 | 0.7056 |
|  | 2 | 1764 (1014–2173) | 1746 (963–2327) | 2079 (1090–3240) |  |  |  |  |  |  |
|  | 3 | 1198 (957–2101) | 1577 (1063–2446) | 2006 (1423–2846) |  |  |  |  |  |  |
|  | 4 | 1398 (970–2378) | 1307 (1054–2439) | 1583 (1067–2310) |  |  |  |  |  |  |
|  | 5 | 1584 (913–2066) | 1436 (957–2672) | 1480 (842–2393) |  |  |  |  |  |  |
|  | 6 | 1893 (1192–2885) | 1318 (719–2271) | 1642 (860–2481) |  |  |  |  |  |  |
|  | 7 | 1166 (966–2508) | 1095 (771–2202) | 1886 (1046–3111) |  |  |  |  |  |  |
|  | 8 | 1386 (695–2331) | 1095 (715–2384) | 1733 (826–2372) |  |  |  |  |  |  |
| Physical Activity Level  (1 = low, 3 = high) | 1 | 2.0 (2.0–2.0) | 2.0 (1.0–2.5) | 2.0 (2.0–3.0) | 0.4595 | 0.6619 | 0.8451 | 0.4640 | 0.2053 | 0.9005 |
|  | 2 | 2.0 (1.5–2.0) | 2.0 (2.0–2.5) | 2.0 (2.0–3.0) |  |  |  |  |  |  |
|  | 3 | 2.0 (2.0–3.0) | 2.0 (2.0–3.0) | 2.0 (2.0–3.0) |  |  |  |  |  |  |
|  | 4 | 2.0 (2.0–2.0) | 2.0 (2.0–2.0) | 2.0 (2.0–2.0) |  |  |  |  |  |  |
|  | 5 | 2.0 (1.5–2.0) | 2.0 (2.0–2.5) | 2.0 (2.0–3.0) |  |  |  |  |  |  |
|  | 6 | 2.0 (2.0–2.5) | 2.0 (2.0–2.0) | 2.0 (2.0–3.0) |  |  |  |  |  |  |
|  | 7 | 2.0 (2.0–2.0) | 2.0 (2.0–2.0) | 2.0 (2.0–3.0) |  |  |  |  |  |  |
|  | 8 | 2.0 (1.0–2.0) | 2.0 (1.4–2.0) | 2.0 (2.0–2.8) |  |  |  |  |  |  |

IQR: Interquartile Range, MET: Metabolic Equivalent of Task, PPC: Pea Protein Concentrate, PPI: Pea Protein Isolate, T: Time, TR: Treatment, T-TR: Time-Treatment Interaction, WPI: Whey Protein Isolate; Significances are indicated in bold (descriptive p-values originating from a non-parametric factorial repeated-measures model with the factors time, treatment, and their interactions); α = 0.05
